# Conserved and Promoter-Specific *Cis*-Regulatory Features of Digestion-Associated Chitinase Genes in *Droseraceae*

**DOI:** 10.3390/ijms27198907

**Published:** 2026-10-07

**Authors:** Martin Jopčík, Monika Danchenko, Ľubomír Harenčár, Jana Libantová

**Affiliations:** Department of Molecular Biology and Biotechnology, Institute of Plant Genetics and Biotechnology, Plant Science and Biodiversity Centre, Slovak Academy of Sciences, Akademická 2, 950 07 Nitra, Slovakia; martin.jopcik@savba.sk (M.J.); monika.danchenko@savba.sk (M.D.); lubomir.harencar@savba.sk (Ľ.H.)

**Keywords:** carnivorous plants, *cis*-element, jasmonic acid, gene regulation, pathogenesis-related proteins, prey digestion, promoter architecture, transcription factor binding site

## Abstract

Carnivorous plants recruit defense-related pathways for prey digestion, yet the *cis*-regulatory architecture underlying their expression remains poorly characterized. In addition to herbivore and fungal pathogen responses, chitinases play an important role in plant carnivory. We analyzed promoters of glycoside hydrolase family 19 (GH19) class I chitinase genes in the Droseraceae family, which are the most abundant chitinases associated with prey digestion. Promoters of newly identified putative class I chitinases found in *Drosera spatulata*, *Drosera tokaiensis*, and *Aldrovanda vesiculosa* were also examined. Core promoter features, *cis*-regulatory elements, transcription factor binding site (TFBS) profiles, *de novo* motifs, and promoter sequence divergence were assessed. Within the sampled promoter set, no single *cis*-element or transcription factor family-compatible pattern was shared by all carnivorous promoters while being absent from *Arabidopsis* reference promoter. Instead, Droseraceae promoters showed candidate ATG-relative positional configurations of shared stress-, defense-, and hormone-responsive elements, including a distal MYC/E-box-compatible–proximal W-box pattern, while an ordered DRE–W-box–ERE configuration occurred in six sundew promoters. The TFBS profiling revealed promoter-specific differences in the representation of loci compatible with bHLH-family models, WRKY, MYB, MYB-related, NAC, and HSF models. Together, these analyses identify candidate promoter-specific patterns and provide a foundation for further broader comparative and functional validation.

## 1. Introduction

Carnivorous plants possess specialized adaptations for capturing, digesting, and absorbing nutrients from animal prey, enabling them to thrive in low-nutrient environments [1]. Species of the Droseraceae family (sundews, the Venus flytrap, and the water-wheel plant) are well known for their active snap and adhesive traps, and for secreting a mixture of hydrolytic enzymes, such as proteases, phosphatases, nucleases, and chitinases, that facilitate prey digestion and subsequent nutrient uptake [1,2,3]. Chitinases enable the degradation of chitin within arthropod exoskeletons by hydrolyzing the ß-1,4-glycosidic linkages between N-acetylglucosamine oligomers [4,5]. Chitinases (EC 3.2.1.14) are divided into glycoside hydrolase (GH) families 18 and 19 based on amino acid sequence similarity [5], and further organized into seven classes according to structure and phylogenetic relationship: classes I, II, IV, VI, and VII comprise GH19, and classes III and V form GH18 [6]. Additionally, in plants, chitinases are recognized as pathogenesis-related (PR) proteins involved in defense against herbivores and fungal pathogens [7]. The PR proteins encompass diverse inducible enzymes and signaling components that contribute to restricting pathogen invasion and progression. In carnivorous plants, duplication and sub-functionalization of GH19 class I chitinases have been proposed: Subclass Ia chitinases are constitutively expressed in the trap leaves for pathogenic response, whereas subclass Ib chitinases are induced in response to prey capture [8,9]. These observations support the hypothesis that carnivorous plants may have co-opted plant defense pathways for digestive processes [10,11]. Previous studies have provided evidence of defense-associated signaling in prey-induced responses in Droseraceae, including jasmonate (JA) signaling in sundew and the Venus flytrap and prey-associated JA accumulation in the water-wheel plant [10,12,13,14]. Several GH19 subclass Ib chitinases have been experimentally associated with prey digestion and represent the most abundant class of chitinases found in the digestive fluid of sundews and the Venus flytrap [2,15,16,17]. Although these enzymes play an important role in prey decomposition, the regulatory elements controlling their gene expression remain poorly understood.

A promoter comprises the DNA sequence 5′ upstream of the open reading frame of a gene, and typically contains a core (flanking the gene’s transcription start site), a proximal regulatory region (adjacent to the core), and a distal enhancer region [18]. Promoter regions determine gene expression by integrating signals from multiple trans-acting transcription factors through specific *cis*-regulatory elements [19]. The composition, abundance, and spatial organization of these elements shape promoter architecture and influence transcriptional responses to tissue-specific, developmental, and environmental signals, especially in stress- and hormone-regulated pathways [20,21]. Earlier findings on the upstream regions of gland-associated genes in the Venus flytrap reported enrichment of WRKY transcription factor expression in activated traps (positive regulator of JA-mediated signaling), suggesting involvement in prey-induced gene regulation [22]. Comparative promoter analysis using computational approaches provides a useful framework for identifying shared regulatory features, promoter-specific patterns, and candidate *cis*-elements for future functional validation [23]. However, no detailed analysis of the regulatory architecture of digestion-associated chitinase gene promoters has been reported in carnivorous plants.

We hypothesized that the sampled GH19 class I chitinase promoters may share defense- and stress-associated regulatory features while differing in motif composition and spatial organization among promoters. Therefore, we performed a comparative bioinformatic analysis of chitinase gene promoter regions from eight species of the Droseraceae family, namely, the lance-leaved sundew (*Drosera adelae*), the forked sundew (*Drosera binata*), the Cape sundew (*Drosera capensis*), the round-leaved sundew (*Drosera rotundifolia*), the spoon-leaved sundew (*Drosera spatulata*), the Tokai sundew (*Drosera tokaiensis*), the Venus flytrap (*Dionaea muscipula*), and the water-wheel plant (*Aldrovanda vesiculosa*). We aimed to identify *cis*-regulatory elements and putative transcription factor binding site profiles, analyze their spatial organization, and relate promoter architecture to candidate regulatory patterns relevant to botanical carnivory.

## 2. Results and Discussion

### 2.1. Digestion-Associated Chitinases in the Droseraceae Family

**Figure 1:**
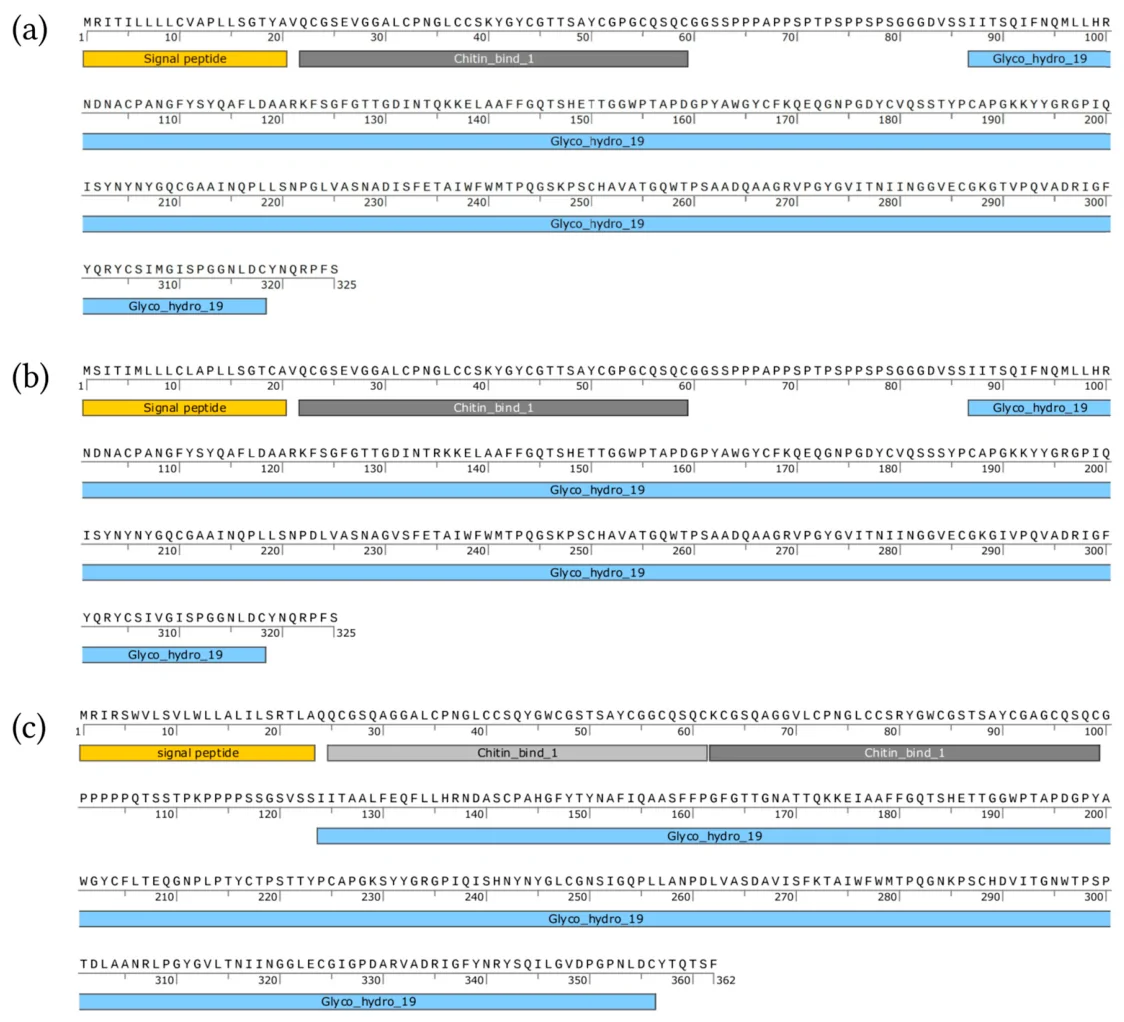
Amino acid sequences of newly identified putative GH19 (class I) chitinases from the spoon-leaved sundew, Tokai sundew, and the waterwheel plant. (**a**) The predicted protein sequence of *D. spatulata* (Ds_00014776-RA) consists of 325 amino acids. (**b**) The predicted protein sequence of *D. tokaiensis* consists of 325 amino acids. (**c**) The predicted protein sequence of *A. vesiculosa* (Av_00023349-RA) consists of 362 amino acids, including two chitin-binding domains. Chitin_bind_1, chitin-binding (recognition) domain in gray rectangles; Glyco_hydro_19, chitinase family (GH) 19 in light blue rectangles; signal peptide in yellow rectangles. Exported from SnapGene (version 8.2.2; SnapGene software).

Previously reported class I chitinase-coding gene sequences from 4 sundew species (*D. adelae*, *D. binata*, *D. capensis*, and *D. rotundifolia*) and the Venus flytrap were initially included in the presented study, with experimentally confirmed roles in prey digestion [2,15,16,17,24]. Additionally, we identified homologs in the translated transcriptomic databases of *D. spatulata* and *A. vesiculosa* [22] using a similarity search against the conserved amino acid sequence of the GH19 glycoside hydrolase domain. Moreover, a putative class I chitinase coding sequence was also detected in the trap leaf transcriptome of *D. tokaiensis*. The deduced protein sequences of the newly identified putative chitinases (Figure 1) showed a highly significant match (*D. spatulata*: query cover 100%, identity 78.2–98.5%; *D. tokaiensis*: query cover 100%, identity 77.2–99.4%; *A. vesiculosa*: query cover 95%, identity 66.9–71.7%; NCBI protein BLAST suite; https://blast.ncbi.nlm.nih.gov/Blast.cgi; accessed on 9 June 2026; identity range represents minimum to maximum identity to digestion-associated chitinases BAR13254.1, QHN63861.1, AZZ09188.1, AMM76171.1, and AHB62682.1) to the other sundew class I chitinases included in the study (Figure S1a).

Subcellular localization analysis predicted signal peptides and association with the secretory pathway for all three putative GH19 class I chitinases (*D. spatulata*: localization probability–extracellular 0.8636, membrane association probability–soluble 0.8810, DeepLoc; Sec/SPI 0.9998, SignalP; *D. tokaiensis*: localization probability–extracellular 0.8414, membrane association probability–soluble 0.8750, DeepLoc; Sec/SPI 0.9998, SignalP; *A. vesiculosa*: localization probability–extracellular 0.8155, membrane association probability–soluble 0.8900, DeepLoc; Sec/SPI 0.9997, SignalP). In addition, the analyzed chitinases lack vacuolar localization signals (C-terminal extensions, CTE; Figure S1b), suggesting extracellular transport [5]. The putative protein sequence of *D. spatulata* is highly similar to the GH19 class I endochitinases of *D. capensis* (320/325 identity) and *D. rotundifolia* (314/325 identity) involved in prey decomposition [16,24]. The Japanese sundew *D. tokaiensis* is a *D. spatulata* × *rotundifolia* hybrid, currently considered a distinct species found in the wild [25]. The translated putative chitinase sequence found in the *D. tokaiensis* transcriptome is almost identical (323/325 identity) to the endochitinase previously characterized from *D. rotundifolia* [16]. Therefore, the analyzed *D. tokaiensis* sequence is considered a likely *D. rotundifolia*-derived homeolog rather than evidence of an independent species-specific evolutionary event. Although both transcribed putative *D. spatulata* and *D. tokaiensis* chitinases show high sequence similarity to previously characterized digestive sundew chitinases and exhibit sequence-based predictions of extracellular targeting, further experimental data are required to demonstrate their digestive function.

To the best of our knowledge, this is the first report of a sequence-based prediction of a GH19 class I chitinase in the trap leaves of *A. vesiculosa*. To date, metallo- and cysteine proteases, acid phosphatase, and esterase have been reported from the digestive fluid of the water-wheel plant [12,26]. Because the prey spectra of *A. vesiculosa* include several taxonomic groups of crustaceans with chitin-rich exoskeletons [27], we hypothesize that the water-wheel plant may also secrete chitinase(s) in the digestive fluid. Strikingly, the translated amino acid chain contained two chitin-binding domains (Figure 1c). Essentially, plant class I chitinases possess only one N-terminal chitin-binding domain; however, rare exceptions exist, such as BjCHI1 isolated from the Indian mustard, *Brassica juncea* [28]. Further gene expression and functional analyses are warranted to experimentally confirm the exceptional nature of the *A. vesiculosa* chitinase predicted in this study.

Phylogenetic analysis placed all eight chitinases of the Droseraceae family within the GH19 class I lineage, distinct from the class IV clade (Figure S1c). The six *Drosera* chitinases formed a well-supported subclass Ib clade (99% bootstrap support), while the broader grouping including *A. vesiculosa* and *D. muscipula* received 70% bootstrap support. The *D. muscipula* subclass Ib chitinase clustered with a subclass Ia paralog from the same species (89% bootstrap support), consistent with diversification within GH19 class I chitinases.

Gene characteristics and protein attributes of all chitinases of the Droseraceae family are summarized in Table 1. The open reading frames exhibited uniform exon distributions, as determined by gene structure analysis. The theoretical isoelectric points (pI) of these eight proteins ranged from 4.30 to 7.79, with the Venus flytrap and the forked sundew chitinase being the most acidic. These proteins’ low aliphatic index (≤ 65%) indicates they are mesophilic; furthermore, all are hydrophilic, as they have negative GRAVY scores.

Available expression and functional evidence for the analyzed genes are summarized in Table S1. Several characterized chitinases are expressed in carnivorous tissues and/or are experimentally associated with digestion, whereas evidence for the newly identified homologs is limited to transcript detection; quantitative expression levels are not directly comparable across species.

### 2.2. Core Promoter Features of the Class I Chitinase-Coding Genes of Carnivorous Droseraceae

The current availability of whole-genome sequences for several carnivorous plant species has facilitated gene identification and the elucidation of their regulation using various bioinformatic tools. We analyzed the promoter sequences of GH19 (class I) chitinase genes (Table 2) from *D. adelae* (P-DaChit), *D. binata* (P-DbChit), *D. capensis* (P-DcChit), *D. rotundifolia* (P-DrChit), *D. spatulata* (P-DsChit), *D. tokaiensis* (P-DtChit), *D. muscipula* (P-DmChit), and *A. vesiculosa* (P-AvChit). Although the accessible promoter sequences differed in total length, the 1500 bp region upstream of the translation start codon (ATG) was used for investigation. All analyzed promoter regions were AT-rich, with AT contents ranging from 60.1% (P-DsChit) to 65.8% (P-AvChit), similar to other plant promoters [29].

Determination of TSSs is pivotal for the identification and characterization of the promoter region, where regulatory elements play key tasks. Experimentally mapped TSSs were available for P-DaChit, P-DbChit, P-DrChit, and P-DmChit, enabling a more reliable assessment of their core promoter architectures. For P-DcChit, P-DsChit, P-DtChit, and P-AvChit, we predicted the TSSs in silico using the Neural Network Promoter Prediction online tool. The prediction revealed that the GH19 chitinase genes of *D. spatulata*, *D. tokaiensis*, *A. vesiculosa*, and *D. capensis* had multiple possible TSSs (ranging from 2 to 4). The highest scores for these promoter regions were above 0.98. In general, the TSSs of all chitinase genes were located between positions −24 and −168 relative to the ATG start codon.

The core promoter element TATA-box (binding of the subunit of the transcription factor IID) was located at positions −39 bp in P-DaChit, −32 bp in P-DrChit, and −25 bp in P-DmChit, with respect to mapped TSSs. Putative TATA-elements were located 32, 25, 25, and 24 bp upstream of the predicted TSSs in P-DtChit, P-DcChit, P-DsChit, and P-AvChit, respectively. In contrast, no TATA-like motif was identified in the expected core promoter region of P-DbChit, relative to the mapped TSS, supporting its classification as a TATA-less promoter [15]. The downstream core promoter element (DPE) plays a function similar to that of the TATA box for TFIID [30]. In our study, the GGTCC motif was found at +28 to +32 relative to the TSS in P-DbChit (DPE consensus sequence RGWYV). The DPE appears to be conserved in several eukaryotic organisms and is located +30 bp of the TSS of many TATA-less promoters [31].

A frequent *cis*-acting element in the core promoter region is the CAAT box (binding site for the nuclear transcription factor Y; NF-Y), which was identified in all analyzed Droseraceae chitinase promoters except P-DbChit, although several degenerate CAAT-like motifs were present in its sequence. The CAAT box acts in two directions [32], contributing to the activation or repression of gene expression through post-translational histone modifications [33].

### 2.3. Putative Cis-Regulatory Motifs Identified in the Chitinase Promoters

The analyzed dataset comprised 10 promoters: eight from the carnivorous Droseraceae family—six *Drosera* spp., together with species from the monotypic genera *Dionaea* (*D. muscipula*) and *Aldrovanda* (*A. vesiculosa*)—and two reference promoters—GH19 chitinase promoter of the Australian carnivorous plant *Cephalotus follicularis* (P-CfChit; Cephalotaceae family of the order Oxalidales) and basic chitinase promoter of *Arabidopsis thaliana* (P-AtChit; Brassicaceae family of the order Brassicales). P-CfChit is the promoter of a chitinase-coding gene, which shows high basal expression in gland-containing pitcher tissues prior to feeding without the involvement of JA-signaling [34], whereas P-AtChit is the promoter of a vacuolar defense-related class I chitinase involved in ethylene/JA-mediated responses and was included as a non-carnivorous reference [9].

The predefined inventory was dominated by stress-, wound-, hormone-, and defense-associated motifs (Table 3). The short, partly degenerate ARE/MYB-overlapping motif was the only category shared by all nine carnivorous promoters and absent from P-AtChit. However, given its limited specificity and the scope of the reference sampling, it was not treated as a discriminatory marker. The canonical CGTCA/TGACG methyl jasmonate (MeJA)-responsive motif occurred in five Droseraceae promoters as well as P-CfChit and P-AtChit. Within the immediate proximal −300 to −1 bp promoter region, it was detected only in P-DmChit (−298) and P-AtChit (−156), providing no shared signature of inducible digestive regulation. Its promoter-wide absence from P-DbChit, P-DrChit, and P-DtChit does not establish jasmonate-independent regulation because jasmonate-associated inputs may involve alternative or combinatorial elements [11,13,35].

The W-box/W-box-like loci were present in all 10 promoters across the full 1500-bp region. Within the immediate proximal region, the Droseraceae promoters contained 15 unique loci, with recorded start coordinates ranging from −278 to −38, whereas neither reference promoter contained such a locus. An exploratory 300-bp sliding-window analysis identified the same Droseraceae vs. reference contrast in 157 of 1201 evaluated windows. This indicates that the contrast was sensitive to the exact position of the 300-bp window. When the proximal region was expanded to −500 to −1 bp, W-box/W-box-like loci in P-CfChit (−491) and P-AtChit (−497 to −493) also fell within it. Thus, the promoters differed in the relative proximity of these loci to the ATG rather than in their promoter-wide presence.

The MYC/E-box-compatible CANNTG motif within the distal −1500 to −1101-bp region occurred in all carnivorous plant promoters but not P-AtChit. Combined with the proximal W-box pattern, it formed a distal CANNTG–proximal W-box combination shared by all Droseraceae promoters; the difference from P-CfChit lay in the proximal W-box component. Because CANNTG is a degenerate consensus, this pattern is compatible with potential bHLH/MYC-associated regulation but does not establish jasmonate-dependent regulation.

Within −500 to −1 bp broader proximal region, six sundew promoters contained at least one ordered DRE–W-box–ERE triplet. The respective start coordinates ranged from −334 to −306 bp for DRE-core loci, −201 to −181 bp for W-box/W-box-like loci, and −129 to −121 bp for ERE loci. No complete triplet occurred in P-DmChit, P-AvChit, P-CfChit, or P-AtChit. This dataset-specific co-occurrence and ordering does not demonstrate conserved spacing or a general feature of carnivorous plants. Other wound- and stress-associated motifs were variably distributed: the WUN-motif was identified only in P-DrChit, P-DtChit, and P-AvChit, whereas WRE3 was detected in P-DbChit and P-DmChit. The *Cephalotus* chitinase promoter also contained a WUN-like variant and WRE3, whereas the non-carnivorous reference P-AtChit lacked both motifs. The STRE motifs were detected in several promoters, but were absent from P-DsChit, P-CfChit, and P-AtChit. These elements may identify candidate promoter-specific inputs related to prey contact, trap stimulation, or elicitor-associated signaling [11,13,36]; however, their occurrence was interpreted with caution due to limited cross-tool support.

Motif occurrence alone cannot establish jasmonate signaling or functional WRKY binding [37,38]. Nevertheless, experimental studies link jasmonate signaling to prey-induced digestive responses in *Drosera* and *Dionaea*, and show prey-induced jasmonate accumulation in *Aldrovanda* [12,13,14]. In *Cephalotus*, feeding did not significantly increase jasmonate levels, proteolytic or phosphatase activities; wounding, however, significantly increased the accumulation of jasmonates, indicating that jasmonate signaling remains functional in a defense context [39].

**Figure 2:**
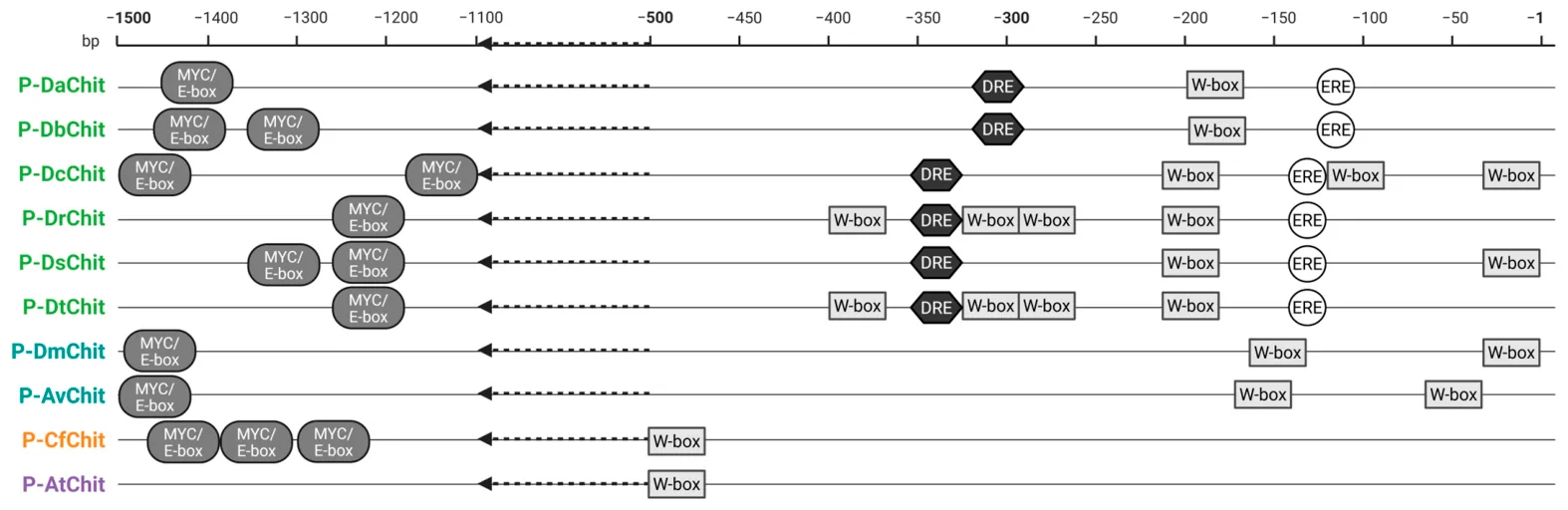
Graphical representation of selected putative *cis*-regulatory elements in GH19 (class I) chitinase promoters of the Droseraceae family (**green** and **teal**), carnivorous pitcher plant *C. follicularis* (**orange**), and the model species *A. thaliana* (**purple**). −300 to −1 bp, immediate ATG-proximal; −500 to −1 bp, broader proximal; −1500 to −1100 bp, distal region. Created in https://BioRender.com (accessed on 6 September 2026).

The promoter-wide occurrence of W-box/W-box-like loci in all investigated sequences and the presence of the CGTCA/TGACG motif across the sampled promoters, including the non-carnivorous *A. thaliana* reference promoter, indicate that these defense-associated sequence features are not restricted to the sampled carnivorous promoters [10,11]. The patterns shared by the Droseraceae promoters may therefore reflect differences in motif combinations and positioning relative to the ATG (Figure 2), consistent with the broader importance of combinatorial *cis*-regulatory control [40]. Similarly, the DRE–W-box–ERE configuration raises a hypothesis of coordinated stress- and ethylene-responsive regulation involving WRKY-compatible sites in the sampled *Drosera* promoters [37,38,41].

Overall, no individual *cis*-element provided a robust discriminator. Instead, the analysis identified two candidate positional configurations: a distal MYC/E-box–proximal W-box pattern shared by the Droseraceae chitinase promoters and a more restricted DRE–W-box–ERE configuration shared by the six sundews (Figure 2). Given the overlap between ancestry and digestive-expression strategy, the use of ATG-referenced coordinates, and the scope of the comparative dataset, these patterns should be regarded as dataset-specific hypotheses for broader comparative and functional testing.

### 2.4. Promoter-Specific Predicted Transcription Factor Binding Site (TFBS)-Family Profiles

**Figure 3:**
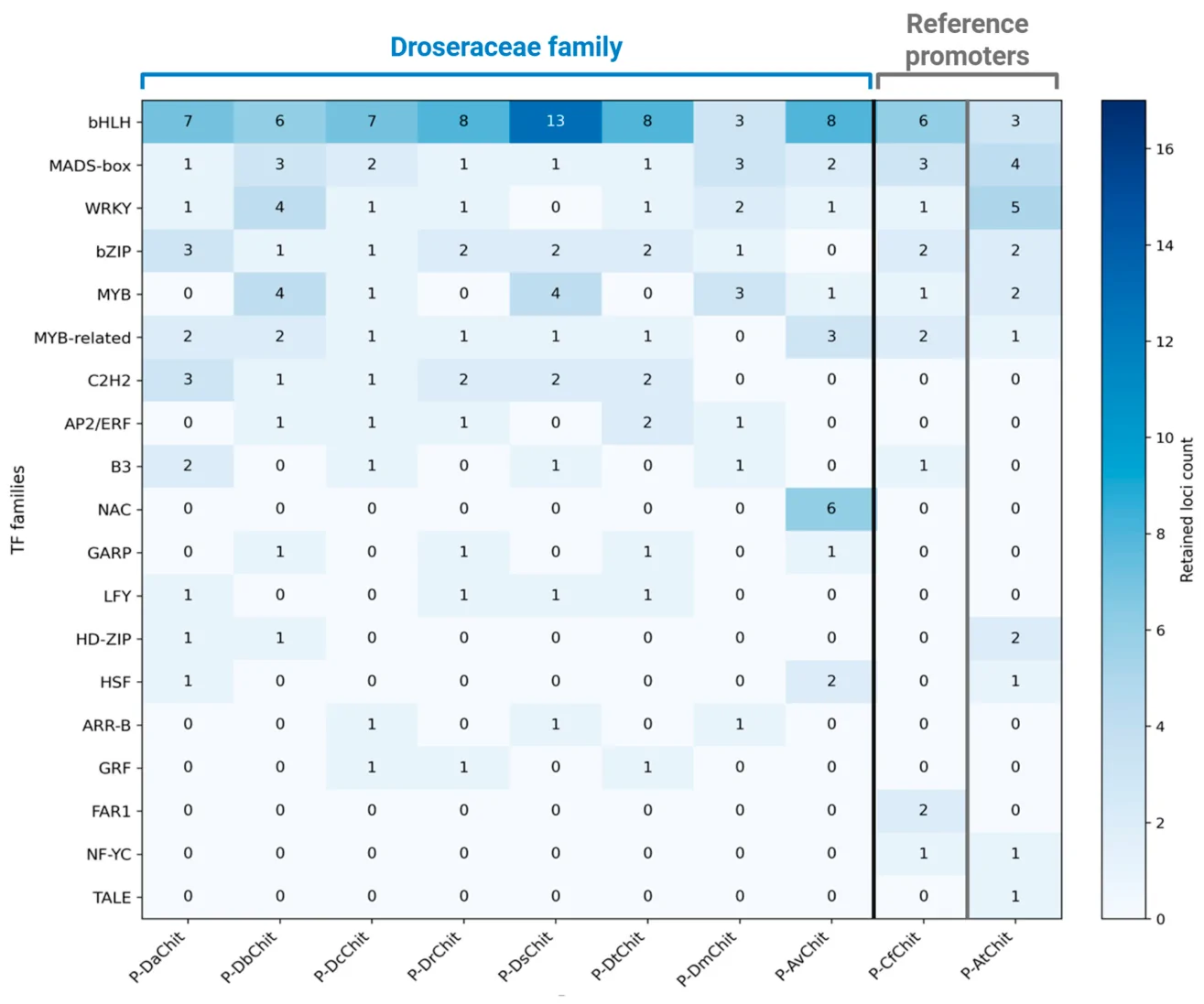
Stringently filtered TF-family-compatible loci across 10 GH19 (class I) chitinase gene promoters. Rows represent TF-family categories; columns represent eight Droseraceae promoters (P-DaChit, *D. adelae*; P-DbChit, *D. binata*; P-DcChit, *D. capensis*; P-DrChit, *D. rotundifolia*; P-DsChit, *D. spatulata*; P-DtChit, *D. tokaiensis*; P-DmChit, *D. muscipula*; P-AvChit, *A. vesiculosa*), *C. follicularis* (P-CfChit) from the Cephalotaceae family, and *A. thaliana* (P-AtChit) from the Brassicaceae family. Cell values and color intensity represent the number of family-level TFBS-compatible loci retained after filtering. TF-family labels indicate compatibility with *A. thaliana*-derived binding models and do not imply experimentally demonstrated TF binding or specific regulatory functions. Exported from Microsoft Excel.

Because short consensus motifs cannot unambiguously identify cognate transcription factors, we compared score-filtered TFBS-family profiles across the chitinase promoters analyzed. Application of the Similar Score threshold (≥0.90) retained 3418 TFBS predictions. Family-level consolidation produced 1288 harmonized loci, of which 1114 were assigned to sequence-specific transcription factor (TF) families. After loci flagged as cross-model-overlapping or low complexity were excluded from this sequence-specific subset, 209 TFBS-compatible loci remained (15–26 per promoter), including 5–9 proximal loci per promoter (Figure 3). These loci indicate sequence compatibility with *A. thaliana*-derived TF-family binding models rather than experimentally demonstrated TF binding or transcriptional regulation [20,42].

The filtered profiles contained several TF-family categories but differed in their relative representation. The bHLH family, including MYC- and PIF-associated models, was the most abundant (69 loci) and occurred in all promoters, followed by MADS-box (21 loci), WRKY (17 loci), bZIP (16 loci), MYB (16 loci), and MYB-related (14 loci). Additional TF families occurred at lower frequencies (Figure 3). MYB and MYB-related predictions were retained as separate TF-family categories. Several of the represented TF families have been broadly associated with stress-, defense-, hormone-, light-, and developmental responses in plants [35,38,41,43,44,45,46,47,48,49,50,51,52,53]. However, because the current predictions are based on heterologous *A. thaliana*-derived binding models, these known associations were not used to assign specific regulatory functions or pathways to the promoters analyzed.

Among the Droseraceae promoters, P-DbChit had the largest WRKY-compatible component, with four WRKY loci accompanied by four MYB, two MYB-related, and six bHLH-compatible loci. P-DmChit showed a mixed profile comprising three MADS-box, three bHLH, three MYB, and two WRKY-compatible loci. P-DsChit contained the largest number of filtered bHLH-compatible loci (13), accompanied by four MYB and one MYB-related loci.

The P-AvChit sequence was distinguished by six NAC- and two HSF-compatible loci, accompanied by eight bHLH, one MYB, and three MYB-related loci. Although the predefined *cis*-element analysis identified a W-box-like variant rather than a canonical W-box in P-AvChit, the filtered TF-family analysis retained one WRKY-compatible locus. This difference is consistent with the distinct motif definitions, scoring thresholds, and filtering procedures used by the two approaches.

P-DaChit had three bZIP- and three C2H2-compatible loci. P-DrChit and P-DtChit had nearly identical filtered profiles, although the *D. tokaiensis* promoter retained one additional AP2/ERF locus. A DREB2A-compatible prediction was present in both of these promoters; in the *D. rotundifolia* sequence, however, the corresponding locus overlapped an HSF prediction and was excluded. The apparent AP2/ERF difference was therefore sensitive to the cross-model-overlap filter and was not interpreted as a clear divergence between their predicted TFBS-family profiles.

Among the two reference promoters, P-CfChit contained two FAR1-compatible loci, whereas P-AtChit contained five WRKY-compatible loci, indicating that WRKY-compatible loci were not confined to carnivorous promoters.

Overall, the predefined *cis*-element and filtered TFBS analyses identified broadly shared sequence features with promoter-specific differences in their representation and organization. No single TF-family-compatible pattern occurred in all nine carnivorous promoters but was absent from P-AtChit. Rather than supporting a shared carnivory-specific motif or TF-family signature, the analyses indicated quantitative differences in the representation of TF-family-compatible loci among the sampled promoters. These patterns should be interpreted with caution because the limited reference sampling does not allow the effects of phylogenetic relatedness to be distinguished from those potentially associated with digestive expression.

**Figure 4:**
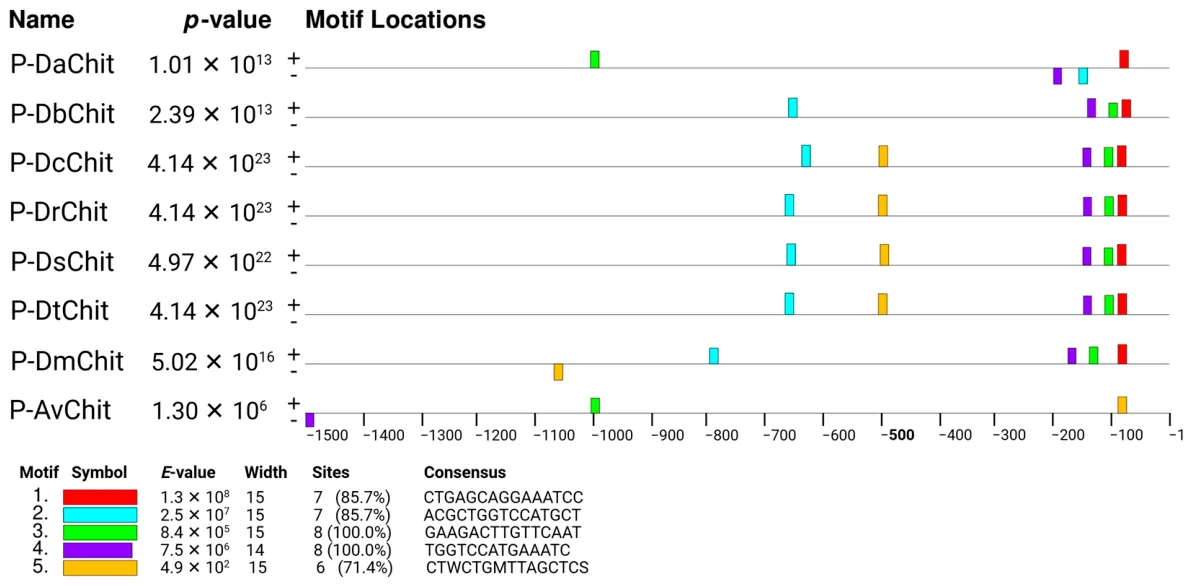
Discovered common candidate motifs of the Droseraceae class I chitinase promoter regions. Block diagram shows the distribution and location of statistically enriched promoter motifs identified by MEME Suite. Various colored rectangles represent distinct motifs. The −500 bp position (in bold) indicates a broader ATG-proximal promoter region.

To examine shared sequence patterns independently of the predefined *cis*-element and TFBS analyses, the Droseraceae chitinase promoters were subsequently analyzed *de novo* using the online motif-based sequence analysis tools, the MEME suite. It was previously reported that genes with similar expression patterns share common motifs in their promoter regions [54]. A common promoter motif can serve as a key signature for a family of co-regulated genes and is usually present in regions where complex protein interactions occur [55]. Therefore, we revealed five candidate motifs (with *E*-value ≤ 0.05) that were shared by the GH19 class I chitinase gene promoter sequences of Droseraceae relative to reference sequences of P-CfChit and P-AtChit (Figure 4). The identified motifs were distributed on both DNA strands and were most densely located at the broader proximal region, between −500 and −50 bp from the ATG start. Motifs 3 and 4 were shared by the eight analyzed promoters, motifs 1 and 2 were detected in *Drosera* and *Dionaea*, but not *Aldrovanda*, and motif 5 was identified in all Droseraceae except the promoters of the two Australian species, P-DaChit and P-DbChit (Figure 4).

Next, the common promoter motifs of Droseraceae class I chitinase genes were compared to the *A. thaliana* DNA motif database (included in JASPAR) to assess their similarity to known regulatory motifs for TFs using the TOMTOM web application. The query motifs returned no statistically significant matches at *q* ≤ 0.05, indicating that similarity to curated TF motifs is limited under the applied conditions. Accordingly, no specific TF identity or regulatory function was assigned to the MEME motifs based on the TOMTOM results. The motifs were retained as candidate sequence patterns for future downstream functional validation.

### 2.5. Promoter Sequence Divergence Analysis

**Figure 5:**
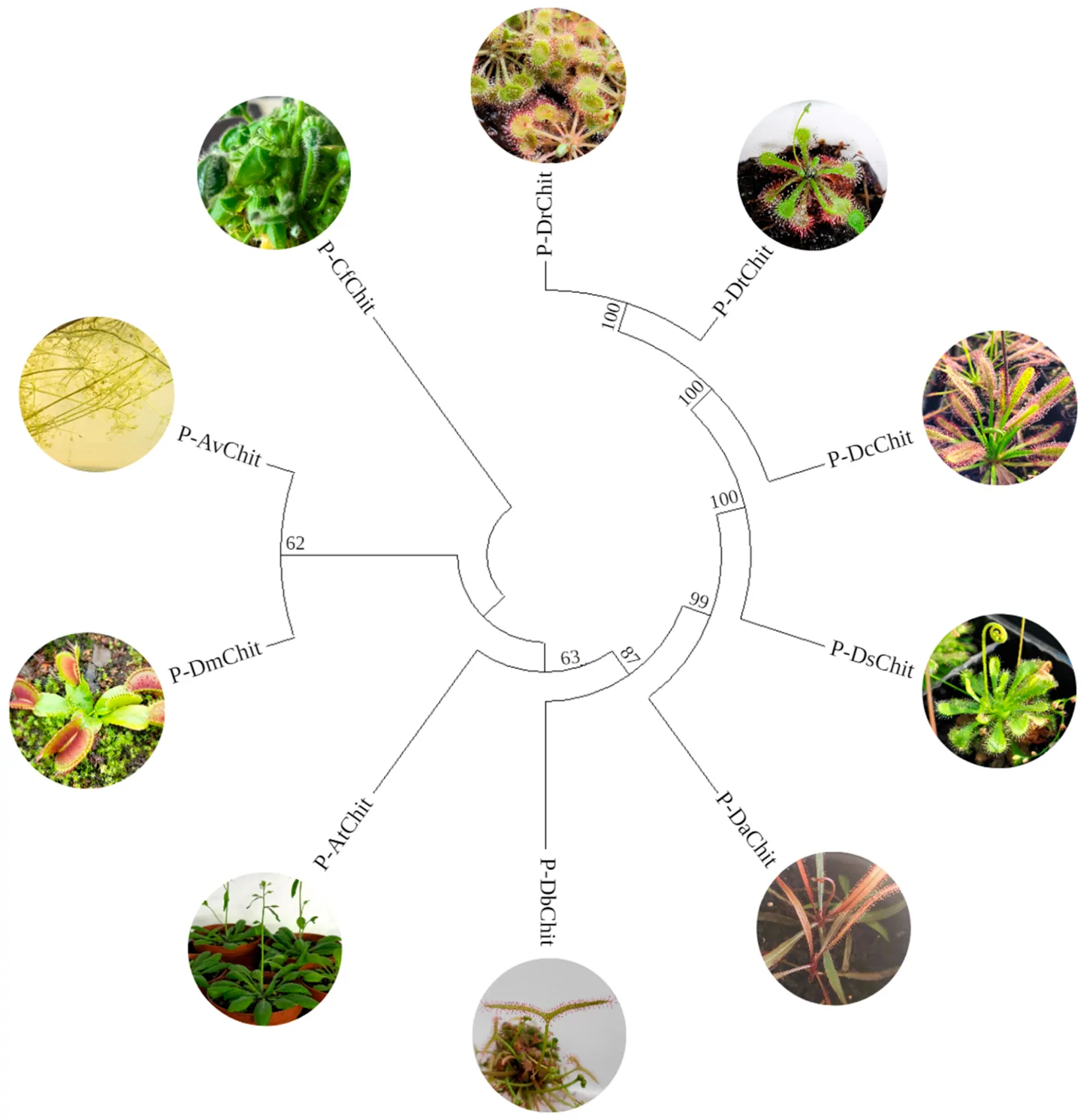
Majority-rule bootstrap consensus tree showing sequence relationships among the GH19 (class I) chitinase promoters of Droseraceae, *C. follicularis*, and *A. thaliana*. The tree was reconstructed using the Neighbor-Joining method from *p*-distances calculated for the 2302-column MUSCLE alignment. Node values indicate bootstrap support based on 1000 replicates; partitions recovered in fewer than 50% of replicates were collapsed. The tree is unrooted, and branch lengths are not proportional to sequence divergence. The tree was exported from MEGA X; Photographs of carnivorous plants by M.D.; Figure created in https://BioRender.com (accessed on 1 September 2026).

The Neighbor-Joining analysis identified a strongly supported core cluster of sundew promoters (Figure 5; Table S2). The P-DrChit and P-DtChit sequences formed the closest pair (*p*-distance = 0.00267; bootstrap support = 100.0%). The close relationship between P-DrChit and P-DtChit mirrors that of their coding sequences and is compatible with *D. rotundifolia* being one progenitor of the amphidiploid *D. tokaiensis* [24]. Of note, recovery of a *D. rotundifolia*-like promoter from *D. tokaiensis* does not exclude the presence of an additional *D. spatulata*-derived homeolog not represented in the analyzed dataset. The chitinase-coding gene promoter of *D. capensis* joined the P-DrChit–P-DtChit pair with 100% bootstrap support and showed *p*-distances of 0.08758 and 0.08902 from P-DrChit and P-DtChit, respectively. *Drosera spatulata* joined this core cluster with 100.0% bootstrap support, followed successively by P-DaChit and P-DbChit with 99.0% and 87.0% support, respectively. The non-carnivorous reference P-AtChit occupied a position adjacent to the broader *Drosera* grouping with 63.0% bootstrap support. The representatives of the two monotypic genera with snap-traps, *Dionaea* and *Aldrovanda*, formed a separate grouping with 62.0% support; however, their relatively high pairwise p-distance (0.57415) indicates that this grouping should not be interpreted as evidence of close sequence similarity. The Australian pitcher plant P-CfChit occupied a separate position in the unrooted topology. Overall, the promoter-sequence analysis provides descriptive evolutionary context for the sampled promoters, but was not used to infer relationships between sequence divergence and TFBS-profile differences.

Taken together, our findings identify promoter-specific configurations of broadly shared stress- and defense-associated sequence features within the sampled dataset. These candidate patterns provide a framework for broader comparative and functional studies of GH19 class I chitinase regulation in carnivorous plants.

## 3. Materials and Methods

### 3.1. Sequence Retrieval and Phylogenetic Analysis of GH19 Chitinases

Promoter sequences of previously reported GH19 class I endochitinase genes from five carnivorous plant species of the Droseraceae family were retrieved from the NCBI GenBank database (accessed on 29 May 2026; https://www.ncbi.nlm.nih.gov/) with the following accession numbers: LC037409.2 gene sequence of *D. adelae* [2]; MN481117.1 gene sequence of *D. binata* [15]; MK093978.1 gene sequence [24] and LIEC02011376.1 whole genome sequence (scaffold 01011500.1; region 18,393–19,687 bp) of *D. capensis* [56]; KU516826.1 gene sequence [16] and BAAGIH010000008.1 whole genome sequence (scaffold 8; region 25,566,514–25,567,513 bp) of *D. rotundifolia*; KF597524.1 gene sequence [17] and JAOWBR010230478.1 whole genome sequence (region 5222–6239 bp) of *D. muscipula* [57].

We retrieved three additional gene sequences of species belonging to the Droseraceae family using publicly available omics datasets. The putative GH19 class I chitinase cDNA of *D. tokaiensis* was identified by screening the leaf transcriptome library GKWH00000000.1 (authors: Procko, C. and Chory, J.) against the *D. rotundifolia* chitinase gene KU516826.1 using the NCBI BLAST suite (accessed on 12 June 2026; https://blast.ncbi.nlm.nih.gov/Blast.cgi). The corresponding promoter sequence of the open reading frame was retrieved from the *D. tokaiensis* genomic library JAOYUI010483990.1 (region 592–2091 bp) [57]. The putative chitinase sequences of *D. spatulata* and *A. vesiculosa* were identified by an identity search for conserved GH19 chitinase domain amino acid motifs (revealed upon alignment with Clustal Omega; Figure S1a) in the translated transcriptome datasets [22] using a text query in TextPad, and the promoter sequences were subsequently retrieved from the corresponding genome regions (both datasets are available at https://www.biozentrum.uni-wuerzburg.de/carnivorom/resources/; accessed on 12 June 2026). To ensure high confidence in the identification of *D. spatulata*, *D. tokaiensis*, and *A. vesiculosa* GH19 class I chitinases, only sequences characterized by all class I chitinase signatures were selected: (i) chitin-binding cysteine-rich domain (Prosite PS00026); (ii) catalytic chitinase 19_1 domain (Prosite PS00773); (iii) catalytic chitinase 19_2 domain (Prosite PS00774); (iv) H-E-T-T motif (Figure S1b) [5]. Additionally, the phylogenetic placement of the eight Droseraceae GH19 class I chitinases was analyzed together with class I paralogs and representative class IV GH19 chitinases from Droseraceae and related taxa (Figure S1c), retrieved from GenBank (accessed on 22 September 2026; https://www.ncbi.nlm.nih.gov/). Amino acid sequences were aligned using Clustal Omega, and the best-fitting substitution model was selected in MEGA X according to the Bayesian Information Criterion (BIC). Maximum-likelihood phylogenetic analysis was performed using the WAG+G model with five discrete Gamma categories (Gamma parameter = 0.8059) and 1000 bootstrap replicates. The analysis included 32 chitinase sequences and 434 aligned positions. The tree was rooted using representative GH19 class IV chitinases as the outgroup.

Promoter sequences of previously reported GH19 chitinase genes of *C. follicularis* (Cfol_v3_22778 and BDDD01001956.1 region 179,247–180,746 bp) [34,58] and *A. thaliana* (HCHIB or At3g12500 and NC_003074.8 region 3,963,985–3,965,484 bp) [59] were included as reference promoters.

For each gene, the 1500 bp upstream region of the translation start codon (ATG) was extracted and used for promoter analysis (Figure S2). This sequence length was selected to standardize comparisons among promoters of different available lengths [40]. The dataset included experimentally supported digestion-associated GH19 class I chitinases and putative homologs identified from transcriptomic and genomic resources. Because comparable quantitative expression data were unavailable across taxa, promoter comparisons were interpreted descriptively without assuming equivalent expression states or regulatory constraints.

### 3.2. Gene Structure Analysis and Characterization of Protein Physicochemical Properties

The intron-exon structure of the predicted chitinase genes was analyzed using the AUGUSTUS gene prediction tool (https://bioinf.uni-greifswald.de/augustus/; accessed on 20 June 2026). Multiple sequence alignment was generated with the Clustal Omega online program (https://www.ebi.ac.uk/jdispatcher/msa/clustalo; accessed on 9 June 2026) and visualized with Jalview version 2.11.5.1 [60]. The ProtParam online tool was used to compute physicochemical properties of proteins (https://web.expasy.org/protparam/; accessed on 9 June 2026). The NCBI Conserved Domain Database (CDD) Search (https://www.ncbi.nlm.nih.gov/Structure/wrpsb-out/wrpsb.cgi; accessed on 9 June 2026) was used to detect protein domains. Prediction of the signal peptide and cleavage site was analyzed using SignalP version 6.0 (https://services.healthtech.dtu.dk/services/SignalP-6.0/; accessed on 9 June 2026). Subcellular localization of the proteins was predicted using DeepLoc version 2.1 (https://services.healthtech.dtu.dk/services/DeepLoc-2.1/; accessed on 9 June 2026).

### 3.3. Determination of Core Promoter Features

Basic promoter sequence characteristics, including the adenine and thymine (AT) content, were determined within the analyzed 1500 bp region using the AT Content Calculator online tool (https://punnettsquare.org/at-content/; accessed on 19 June 2026). The TSSs were predicted using the Neural Network Promoter Prediction (NNPP) online tool version 2.2 (https://www.fruitfly.org/seq_tools/promoter.html; accessed on 19 June 2026), with the minimum standard predictive score of 0.8. For promoter regions containing multiple TSS, the TSS with the highest prediction score was selected. The TATA box (TATAWAW consensus [61]) and CAAT box (CCAAT consensus [32]) were identified using the PlantCARE plant *cis*-acting regulatory element online database (https://bioinformatics.psb.ugent.be/webtools/plantcare/html/; accessed on 22 June 2026). Promoters were classified as TATA-plus (TATA box consensus present with experimentally confirmed TSS), TATA-less (TATA box consensus absent but experimentally confirmed TSS), or putative TATA-plus (TATA box consensus present with in silico predicted putative TSS). Canonical CCAAT boxes were identified as exact matches to the consensus sequence CCAAT. CAAT-like variants, such as CAAAT, CCAAC, and GCAAT, were recorded separately.

### 3.4. In Silico Identification and Harmonization of Cis-Regulatory Motifs

The analyzed dataset comprised eight chitinase promoters from the Droseraceae family and two reference promoters: the GH19 chitinase promoter of *C. follicularis* (P-CfChit) and the class I chitinase promoter of *A. thaliana* (P-AtChit). The sequences were screened for a predefined set of putative *cis*-regulatory motifs associated with jasmonate, wound, and defense responses; ethylene, anaerobic, and general stress responses; DREB/AP2-related regulation; MYB- and MYC/E-box-associated regulation; auxin and low-temperature responses; and ACGT/ABRE-like regulation. The selected AATAGTT motif was retained without a specific functional assignment.

The analyses were performed using PlantCARE (https://bioinformatics.psb.ugent.be/webtools/plantcare/html/; accessed on 3 August 2026), PlantPAN version 4.0 (https://plantpan.itps.ncku.edu.tw/plantpan4/promoter_analysis.php; accessed on 3 August 2026), and New PLACE (https://rapdb.dna.naro.go.jp/PLACE/?action=newplace; accessed on 3 August 2026) databases. All searches were conducted with the default database settings. For each promoter, the database-specific annotation, the matched sequence, the strand orientation, and the ATG-relative position of each hit were recorded. Calls representing the same predefined motif across databases were harmonized into common motif categories based on matched sequences and consensus definitions, rather than annotation names alone. Both DNA strands were considered.

A hit was classified as an exact match only when its sequence matched the predefined consensus. Related but non-identical matches were retained only when supported by the corresponding database annotation and designated as motif variants. Database-annotated W-box variants were retained only when they contained the relevant W-box core and were annotated as WRKY-related elements. Exact W-boxes and retained W-box-like variants were combined within the W-box/W-box-like category. Non-identical WUN-related calls were labeled as WUN-like variants. ACGT-containing variants were retained when they contained the predefined ACGT/G-box-like core and their database annotations supported an ABRE-, G-box, bZIP-, or related light/stress-associated classification.

Motif coordinates were expressed relative to the translation start codon, defined as +1; thus, positions 1–1500 of each input sequence corresponded to −1500 to −1 bp. Single values in Table 3 indicate the ATG-relative coordinate of the first nucleotide of an individual harmonized motif locus in the submitted 5′–3′ promoter sequence, irrespective of the motif strand orientation. Within each promoter and harmonized motif category, overlapping calls representing the same physical locus—including opposite-strand duplicates and nested database annotations—were collapsed into one unique locus. Coordinate ranges in Table 3 represent the combined span of overlapping database calls consolidated at the same locus. Because experimentally determined transcription start sites were unavailable for all promoters, positional comparisons were performed relative to the ATG and were not interpreted as exact distances from TSS.

For descriptive positional comparisons, the 1500-bp promoter region was divided into five non-overlapping 300-bp regions: −1500 to −1201; −1200 to −901; −900 to −601; −600 to −301; and −300 to −1 bp. The −300 to −1 bp region was designated the immediate ATG-proximal region, whereas −500 to −1 bp was evaluated separately as a broader proximal region. A motif category was scored as present in a region when at least one unique harmonized locus overlapped it.

As an exploratory boundary-sensitivity analysis, a 300-bp window was shifted in 1-bp increments from −1500/−1201 to −300/−1, yielding 1201 windows. W-box/W-box-like presence was scored when at least one unique harmonized locus overlapped the evaluated window. A complete contrast required W-box/W-box-like presence in all eight Droseraceae promoters and absence from both *C. follicularis* and *A. thaliana* promoters within the same window. No window-specific inferential tests were performed.

### 3.5. Prediction and Harmonization of Transcription Factor-Binding Sites and Screening for Common Promoter Motifs

The same 10 promoter sequences (Section 3.4) were analyzed using the Promoter analysis function of PCBase version 3.0 (https://pcbase.itps.ncku.edu.tw/pcbase3/promoter_analysis/; accessed on 6 August 2026), a companion resource of the PlantPAN version 4.0 platform. Because species-specific TF-binding models were unavailable for the carnivorous plants analyzed here, *A. thaliana* was used as the source organism for TF-binding models. Predictions were therefore interpreted as sequence compatibility with *A. thaliana*-derived TF-binding models, rather than as evidence of TF binding in carnivorous plants. Only TFBS predictions with a Similar Score ≥ 0.90 were retained. Predicted TF identifiers were manually standardized to canonical *A. thaliana* names and assigned to TF families primarily according to PlantTFDB version 5.0 classification [62]. Subsequent analyses were conducted at the TF-family level. Non-sequence-specific regulatory proteins were excluded from the TF-family counts, but retained during cross-model overlap screening. The original PCBase coordinates from 1 to 1500 were converted to positions relative to the ATG start codon, such that positions 1 and 1500 corresponded to −1500 and −1 bp, respectively. For each prediction, the matched core was defined by the uppercase sequence in the PCBase Hit Sequence output, and its core region was inferred from the reported position and core length.

Within each promoter, predictions assigned to the same TF family were merged into a single family-level locus when their inferred core regions overlapped by at least one nucleotide. Merging was transitive, meaning that predictions connected through a chain of pairwise overlaps were treated as one locus. A locus was flagged as cross-model-overlapping when it overlapped a locus assigned to a different TF family or regulatory class, including a non-sequence-specific regulatory model.

An individual core was classified as low complexity if it was shorter than five nucleotides or if a single nucleotide accounted for at least 80.0% of its sequence. A merged family-level locus was classified as low complexity only when all its supporting predictions had low-complexity cores. The stringently filtered TFBS-compatible dataset used for promoter-level comparisons and the heat map excluded loci flagged as cross-model-overlapping or low complexity. A TFBS locus was classified as proximal if any part of its region overlapped the region from −500 to −1 bp relative to the ATG.

For visualization and promoter-level comparisons, the stringently filtered loci were summarized according to the standardized TF-family annotations of the underlying *A. thaliana*-derived binding models, primarily following PlantTFDB version 5.0 classification. The MYB and MYB-related predictions were retained as separate family-level categories, as were the other PlantTFDB TF families. The MYC2-, MYC3-, and PIF-associated predictions were summarized within the bHLH family because the corresponding models are classified within this family. TF-family labels, therefore, refer to the annotation of the underlying prediction models and do not imply that a matched promoter sequence uniquely identifies a particular TF, TF subgroup, or functional pathway. Because DNA-binding preferences may overlap among TFs within and across families, the retained loci were interpreted only as sequence regions compatible with the corresponding TF-family models.

Common (novel) motifs in the promoter regions of the Droseraceae GH19 class I chitinase genes were identified by online web-based analysis, MEME (Multiple Em for Motif Elicitation) suite version 5.5.9 (https://meme-suite.org/meme/tools/meme; accessed on 4 August 2026) with a maximum of five motifs. Enriched motifs in Droseraceae promoters were discovered in discriminative mode using P-CfChit and P-AtChit as the second (control) set. Other parameters of motif discovery included: ZOOPS model; motif width = 6–15 bp; *E*-value ≤ 0.05. The significant motifs 1–4, which occurred in the majority of promoter regions, were forwarded to TOMTOM (Motif Comparison Tool) web server (https://meme-suite.org/meme/tools/tomtom; accessed on 4 August 2026) for identification of candidate TF-binding motif similarities in the JASPAR CORE Plants and *Arabidopsis* database (https://jaspar.elixir.no/; accessed on 4 August 2026) with significance threshold *q*-value ≤ 0.05.

### 3.6. Promoter Sequence Divergence Analysis

The Droseraceae and reference promoter sequences (*n* = 10) were aligned using the MUSCLE algorithm implemented in MEGA X [63]. The resulting multiple-sequence alignment comprised 2302 alignment columns. Pairwise sequence distances were calculated using the *p*-distance method and expressed as the number of nucleotide differences per site. Both transitions and transversions were included, substitution rates among sites were assumed to be uniform, and substitution patterns were assumed to be homogeneous among lineages. Sites containing gaps, missing data, or ambiguous positions were excluded separately for each sequence pair using the pairwise-deletion option.

A Neighbor-Joining tree was constructed from the *p*-distance matrix [64]. A majority-rule bootstrap consensus tree was generated from the 1000 replicates, and partitions recovered in fewer than 50% of the bootstrap replicates were collapsed. The resulting tree was presented as an unrooted representation of promoter-sequence relationships. The pairwise *p*-distances are provided in Table S2.

## Figures and Tables

**Table 1:** Gene structure characteristics and physicochemical properties of the digestion-associated and putative GH19 class I chitinases in the Droseraceae family.

Species	ORF (bp)	Intron/Exon	CDS (bp)	AA Residues	MW (kDa)	pI	Aliphatic Index	GRAVY
*Drosera adelae*	1335	2/3	966	321	33.9	5.88	56.95	−0.285
*Drosera binata*	1276	2/3	966	321	33.8	5.35	61.21	−0.212
*Drosera capensis*	1662	2/3	978	325	34.2	7.79	58.89	−0.212
*Drosera rotundifolia*	1665	2/3	978	325	34.0	7.49	58.31	−0.209
*Drosera spatulata*	1692	2/3	978	325	34.1	7.34	58.62	−0.222
*Drosera tokaiensis*	1625	2/3	978	325	34.0	7.33	59.51	−0.187
*Dionaea muscipula*	1558	2/3	945	314	33.4	4.30	57.26	−0.282
*Aldrovanda vesiculosa*	1405	2/3	1089	362	38.3	7.70	62.35	−0.127

AA, amino acid; CDS, coding sequence; GRAVY, grand average of hydropathy; MW, molecular weight; ORF, open-reading frame; pI, isoelectric point.

**Table 2:** Core promoter features of GH19 class I chitinase genes analyzed in carnivorous plants.

Features	AT Content (%)	TSS (Method or Score)	TATA Box Status	CCAAT Box	CAAT-Like Motifs
P-DaChit	64.7	−24 (RNA-seq)	TATA-plus	Present	Present
P-DbChit	64.5	−168 (RACE)	TATA-less	Absent	Present
P-DcChit	65.5	−42 (0.99)	Putative TATA-plus	Present	Present
P-DrChit	65.3	−41 (RACE)	TATA-plus	Present	Present
P-DsChit	60.1	−42 (0.99)	Putative TATA-plus	Present	Present
P-DtChit	65.4	−41 (0.99)	Putative TATA-plus	Present	Present
P-DmChit	62.3	−42 (RACE)	TATA-plus	Present	Present
P-AvChit	65.8	−38 (0.98)	Putative TATA-plus	Present	Present

RACE, rapid amplification of cDNA ends; RNA-seq, next-generation RNA sequencing; TSS, transcription initiation site (values represent the highest prediction scores for promoter sequences having multiple TSSs, as determined by the Neural Network Promoter Prediction tool). Promoter sequences: P-DaChit, *D. adelae*; P-DbChit, *D. binata*; P-DcChit, *D. capensis*; P-DrChit, *D. rotundifolia*; P-DsChit, *D. spatulata*; P-DtChit, *D. tokaiensis*; P-DmChit, *D. muscipula*; P-AvChit, *A. vesiculosa*.

**Table 3:** Complete distribution of harmonized putative *cis*-regulatory motifs in the GH19 (class I) chitinase promoters from Droseraceae, *C. follicularis*, and *A. thaliana*.

Consensus Motif	Harmonized Element	Functional Category	P-DaChit	P-DbChit	P-DcChit	P-DrChit	P-DsChit	P-DtChit	P-DmChit	P-AvChit	P-CfChit	P-AtChit
CGTCA/ TGACG	MeJA- responsive motif	MeJA/jasmonate response	−886	•	−1244	•	−1241; −939	•	−1402; −348; −298	−1168; −950	−639	−156
TTGAC[C/T]	W-box/ W-box-like	WRKY/defense/ elicitor response	−1212–1199; −198–185	−1352–1348; −1060–1056; −1037–1033; −979–975; −181–177	−1498; −814; −201; −121; −38	−1084; −1012; −838; −710; −392; −312; −278; −200	−1431; −887; −201; −38	−1084; −1012; −838; −710; −392; −312; −278; −200	−674; −598; −460; −146; −38	−1247; −1032; −1002; −606; −580; −556; −166; −53	−1291; −1269; −1120; −1060; −576; −491	−1431; −1207; −927–923; −791; −733; −600; −537–533; −497–493
AAATTACTA	WUN-motif	Wound/biotic stress response	•	•	•	−270	•	−270	•	−802	−1085 v	•
CCACCT	WRE3	Wound/defense response	•	−266	•	•	•	•	−275	•	−804	•
ATTTCATA	ERE	Ethylene response	−125	−121	−129	−128	−129	−128	•	•	•	−805
AAACCA/ WAACCA	ARE/MYB-overlapping site	Hypoxia-associated/MYB-overlapping regulation	−670	−1287; −1100; −894; −870; −243	−1385; −1113; −934; −617; −341	−971; −646; −340	−643; −337	−971; −646	−1017; −49	−1460 −808; −635; −574	−1197; −807; −721	•
AGGGG	STRE	General stress response	−764	−70	−949	−1382; −986	•	−1382; −986	−1457; −1386; −389; −66	−1288 −979	•	•
[AG]CCGAC	DRE core	DREB/AP2-related abiotic stress response	−307	−306	−334	−333	−330	−333	−1031	−597 v	−327	•
CNGTTR	MYB core	MYB-related	−405	−518; −308	−828; −336	−534; −399; −335; −302; −14	−1213; −1095; −793; −396; −332	−534; −399; −335; −302; −14	−100	−1367; −1144	−1284; −1165; −463; −404; −333	−1264
TAACNA	TAACNA motif	MYB/auxin-related	−1065; −861; −752; −483; −259	−1127; −1100; −894; −870; −782; −243	−1113; −617; −398; −271; −14	−865; −646; −588	−1458; −1297; −1254; −1253; −643; −585; −271; −14	−865; −646; −588	−715; −494; −318	−1018; −759; −475	−1197; −1176; −1078; −807; −726; −419	−1158; −895; −586; −294; −78
CANNTG	MYC/E-box site	MYC/E-box-related	−1406	−1428; −1308; −1004; −874; −677; −507; −341; −200; −80	−1483; −1123; −788; −767; −356; −102	−1202; −913; −793; −734; −355	−1321; −1213; −352; −102	−1202; −913; −793; −734; −355	−1466; −858; −365; −100	−1489; −703; −639; −500; −77	−1430; −1362; −1253; −53	−672
TGTCTC	AuxRE/ARF-binding site	Auxin response	•	−580	•	•	•	•	•	•	•	−677
AATAGTT	AATAGTT motif	Unclassified selected motif	−1278	•	−273	•	•	−273	•	•	•	•
CCGAAA	LTR	Low-temperature response	−250	•	•	•	•	−764	−775	−775	−712	−973
CACGTG/ ACGT- containing	ACGT/ABRE-like motif	ABA-, light-, and stress-associated bZIP regulation	−1473; −1386	−1123	•	−1228	−1228; −877; −817; −25	−1157; −1110; −855; −808	•	•	−1486	−908; −825; −702; −569; −548; −389; −339

v, motif variant; Bullet indicates absence of the motif; Single coordinates indicate the ATG-relative position of the first nucleotide of an individual harmonized motif locus; Coordinate ranges represent the combined span of overlapping database calls consolidated at the same locus and may therefore exceed the length of the consensus motif; Promoter sequences: P-DaChit, *D. adelae*; P-DbChit, *D. binata*; P-DcChit, *D. capensis*; P-DrChit, *D. rotundifolia*; P-DsChit, *D. spatulata*; P-DtChit, *D. tokaiensis*; P-DmChit, *D. muscipula*; P-AvChit, *A. vesiculosa*; P-CfChit, *C. follicularis*; P-AtChit, *A. thaliana*.

## Data Availability

The original contributions presented in this study are included in the article/Supplementary Material. Further inquiries can be directed to the corresponding author.

## Supplementary Materials

The following supporting information can be downloaded at: https://www.mdpi.com/article/10.3390/ijms27198907/s1.

## Abbreviations

The following abbreviations are used in this manuscript:

ABA	Abscisic acid
ABRE	Abscisic acid-responsive element
AP2/ERF	Ethylene-responsive element binding factor
ARE	Auxin response element
ARR-B	Type-B *Arabidopsis* response regulator
AuxRE/ARF	Auxin response element/Auxin response factor
B3	Third basic region domain
bHLH	Basic helix-loop-helix
bZIP	Basic leucine zipper
C2H2	Cysteine 2-Histidine 2
CTE	C-terminal extension
DPE	Downstream core promoter element
DREB2A	Dehydration-responsive element-binding protein 2A
E-box	Enhancer-box
ERE	Ethylene response element
FAR1	FAR-RED-IMPAIRED RESPONSE 1
GARP	Golden2-ARRB (Type-B *Arabidopsis* response regulator)-Psr1 (Phosphate starvation response 1)
GH19	Glycoside hydrolase family 19
GRAVY	Grand average of hydropathy
GRF	Growth-regulating factor
HD-ZIP	Homeodomain-leucine zipper
HSF	Heat shock factor
JA	Jasmonate
LFY	LEAFY
LTR	Low-temperature response
MeJA	Methyl jasmonate
MADS-box	MCM1 (Minichromosome maintenance 1)- AGAMOUS- DEFICIENS-SRF (Serum response factor)
MEGA	Molecular evolutionary genetics analysis
MEME	Multiple em for motif elicitation
MUSCLE	Multiple sequence comparison by log-expectation
MYB	Myeloblastosis transcription factor family
MYC	Myelocytomatosis-type basic helix-loop-helix transcription factor
NAC	NAM (No apical meristem)-ATAF1/2 (*A. thaliana* transcription activator factor 1/2)-CUC2 (Cup-shaped cotyledon 2)
NF-YC	Nuclear transcription factor Y subunit gamma
PIF	PHYTOCHROME-INTERACTING FACTOR
PR	Pathogenesis-related
Sec/SPI	Secretory signal peptides transported by the Sec translocon and cleaved by Signal Peptidase I
STRE	Stress response element
TALE	Three-amino-acid loop extension
TFIID	Transcription factor IID
TF	Transcription factor
TFBS	Transcription factor binding site
TSS	Transcription initiation site
WRE3	Wound-responsive element 3
WRKY	W (tryptophan) R (arginine) K (lysine) Y (tyrosine)
WUN-motif	Wound-responsive motif
ZOOPS	Zero-or-one-occurrence-per-sequence
