# Supplementary material for: Conserved and Promoter-Specific Cis-Regulatory Features of Digestion-Associated Chitinase Genes in Droseraceae
Source: Int J Mol Sci. 2026 Oct 7;27(19):8907. doi: 10.3390/ijms27198907 (PMC13665433; doi:10.3390/ijms27198907)
Supplement: Supplementary file 1 [file ijms-27-08907-s001.zip › ijms-4573619-supplementary.pdf]

Conserved and promoter-specific *cis*-regulatory features of digestion-associated chitinase genes in Droseraceae

Martin Jopčík [†], Monika Danchenko [†], Ľubomír Harenčár, and Jana Libantová *

Institute of Plant Genetics and Biotechnology, Plant Science and Biodiversity Centre, Slovak Academy of Sciences, Akademicka 2, P. O. Box 39A, 950 07 Nitra, Slovak Republic

* Correspondence: jana.libantova@savba.sk (J.L.)

[†] These authors contributed equally to this work.

SUPPLEMENTARY MATERIAL

Figure S1: Multiple sequence alignment of GH19 (class I) chitinases included in the study

Figure S2: Nucleotide sequences and their accession numbers of the analyzed GH19 chitinase gene promoters

Table S1: Available expression and functional evidence for the analyzed Droseraceae GH19 class I chitinases

Table S2: Pairwise uncorrected p-distances among the analyzed 1,500-bp chitinase promoter sequences

(a)

```
Av_00023349-RA MRIRSWVLSVLWLLALILSRTLAQQCGSQAGGALCPNGLCCSQYGWCGSTSAYCGGCQSQCKCGSQAGGVLCPNGLCCSRYGWCGSTSAYCGA 93
Dm_AHB62682.1  --MRNWVLL-LLCISPFFSRTLAEQCGSQAGGALCP-------------------------------------NGLCCSQYGWCGTTSAYCGA 53
Db_QHN63861.1  --MRICILL-LLCVSLFLSGALAEQCGYQAGGAVCP-------------------------------------NGLCCSQYGYCGTTSAYCGS 53
Da_BAR13254.1  -MMKISLLL-LLFVVPLLSGTSAQQCGYQAGGALCP-------------------------------------NGLCCSKYGYCGTTSAYCGD 54
Dr_AMM76171.1  --MSITIML-LLCLAPLLSGTCAVQCGSEVGGALCP-------------------------------------NGLCCSKYGYCGTTSAYCGP 53
Dt_ChitI       --MSITIML-LLCLAPLLSGTCAVQCGSEVGGALCP-------------------------------------NGLCCSKYGYCGTTSAYCGP 53
Dc_AZZ09188.1  --MRITVLL-LLCVAPLLSGTYAVQCGSEVGGALCP-------------------------------------NGLCCSKYGYCGTTSAYCGP 53
Ds_00014776-RA --MRITILL-LLCVAPLLSGTYAVQCGSEVGGALCP-------------------------------------NGLCCSKYGYCGTTSAYCGP 53

Av_00023349-RA GCQSQCGPPPPPQTSSTPK--PP-PPSSGSVSSIITAALFEQFLLHRNDASCPAHGFYTYNAFIQAASFFPGFGTTGNATTQKKEIAAFFGQT 183
Dm_AHB62682.1  GCQSQCGGSS-----------PPSPSGGGDVGSIVTNEIFNQMLLHRNDNACPANGFYTYNAFIEAARSFPGFGTTGDVNTQRKELAAFFGQT 135
Db_QHN63861.1  GCQSQCGGGSPPPSPP----LPPSPSGGGDVSSIITSQIFNEMLLHRNDNACPANGFYTYDAFIQAARSFPGFGTTGDINTRKKELAAFFGQT 142
Da_BAR13254.1  GCQSQCGGSSPPPAPP----SPPSPSGGGDVSSIITSQIFNQMLLHRNDNACPANGFYSYQAFINAARKFSGFGTTGDTNTRKNELAAFFGQT 143
Dr_AMM76171.1  GCQSQCGGSSPPPAPPSPTPSPPSPSGGGDVSSIITSQIFNQMLLHRNDNACPANGFYSYQAFLDAARKFSGFGTTGDINTRKKELAAFFGQT 146
Dt_ChitI       GCQSQCGGSSPPPAPPSPTPSPPSPSGGGDVSSIITSQIFNQMLLHRNDNACPANGFYSYQAFLDAARKFSGFGTTGDINTRKKELAAFFGQT 146
Dc_AZZ09188.1  GCQSQCGGSSPPPAPPSPTPSPPSPSGGGDVSSIITSQIFNQMLLHRNDNACPAHGFYSYQAFLDAARKFTGFGTTGDINTRKRELAAFFGQT 146
Ds_00014776-RA GCQSQCGGSSPPPAPPSPTPSPPSPSGGGDVSSIITSQIFNQMLLHRNDNACPANGFYSYQAFLDAARKFSGFGTTGDINTQKKELAAFFGQT 146

Av_00023349-RA SHETTGGWPTAPDGPYAWGYCFLTEQGNPLPTYCTPSTTYPCAPGKSYYGRGPIQISHNYNYGLCGNSIGQPLLANPDLVASDAVISFKTAIW 276
Dm_AHB62682.1  SHETTGGWPTAPDGPYAWGYCFKQEQGNP-PSYCEPSTAYPCAPGKSYYGRGPIQISYNYNYGQCGDSIGQPLLANPDLVADDALISFETAIW 227
Db_QHN63861.1  SHETTGGWPTAPDGPYAWGYCFKEEQGNP-GSYCVASSTYPCAPGKKYYGRGPIQISYNYNYGQCGAAINQPLLANPDLVASNADISFETAIW 234
Da_BAR13254.1  SHETTGGWSTAPDGPYAWGYCFKQEQGNP-GDYCAPSSTYPCAPGQKYYGRGPIQISYNYNYGLCGAAINQPLLSNPGLVASDADISFETAIW 235
Dr_AMM76171.1  SHETTGGWPTAPDGPYAWGYCFKQEQGNP-GDHCVQSSSYPCAPGKKYYGRGPIQISYNYNYGQCGAAINQPLLSNPDLVASNAGVSFETAIW 238
Dt_ChitI       SHETTGGWPTAPDGPYAWGYCFKQEQGNP-GDYCVQSSSYPCAPGKKYYGRGPIQISYNYNYGQCGAAINQPLLSNPDLVASNAGVSFETAIW 238
Dc_AZZ09188.1  SHETTGGWPTAPDGPYAWGYCFKQEQGNP-GDYCVQSSTYPCAPGKKYYGRGPIQISYNYNYGQCGAAINQPLLSNPDLVASNADVSFETAIW 238
Ds_00014776-RA SHETTGGWPTAPDGPYAWGYCFKQEQGNP-GDYCVQSSTYPCAPGKKYYGRGPIQISYNYNYGQCGAAINQPLLSNPGLVASNADISFETAIW 238

Av_00023349-RA FWMTPQGNKPSCHDVITGNWTPSPTDLAANRLPGYGVLTNIINGGLECGIGPDARVADRIGFYNRYSQILGVDPGPNLDCYTQTSF-      362
Dm_AHB62682.1  FWMTPQGDKPSCHDVITGNWSPSSADQAAGRLPGYGVITNIINGGVECGKGQDASVADRIGFYTRYCNILGVNPGNNLDCYNQAPFS      314
Db_QHN63861.1  FWMTPQGNKPSCHAVITGQWSPSSADQAAGRVPGYGVITNIINGGIECGKGQNAEAANRIGFYERYCSLLGISPGNNLDCNNQRPFS      321
Da_BAR13254.1  FWMTPQGNKPSCHAVATGQWNPSSADQAAGRVPGYGVITNIINGGVECGQGEKAEVADRIGFYQRYCSIFGISPGQNLDCYNQRPFS      322
Dr_AMM76171.1  FWMTPQGSKPSCHAVATGQWTPSAADQAAGRVPGYGVITNIINGGVECGKGTVPQVADRIGFYQRYCSIVGISPGGNLDCYNQRPFS      325
Dt_ChitI       FWMTPQGSKPSCHAVATGQWTPSAADQAAGRVPGYGVITNIINGGVECGKGIVPQVADRIGFYQRYCSIVGISPGGNLDCYNQRPFS      325
Dc_AZZ09188.1  FWMTPQGSKPSCHAVATGQWTPSVADQAAGRVPGYGVITNIINGGVECGKGTVPQVADRIGFYQRYCSIMGIAPGGNLGCYNQRPFS      325
Ds_00014776-RA FWMTPQGSKPSCHAVATGQWTPSAADQAAGRVPGYGVITNIINGGVECGKGTVPQVADRIGFYQRYCSIMGISPGGNLDCYNQRPFS      325
```

(b)

```
Cf_GAV79313.1     ----------MVPVRKYILT--IVLALVLAGAVPKNVV----------------------      26
At_NP_566426.2    MPPQKENHRTLNKMKTN-LFLFLIFSLLLSLSSAEQCGRQAGGALCPNGL----------      49
Av_00023349-RA    -----------MRIRSWVLSVLWLLALILSRTLAQQCGSQAGGALCPNGLCCSQYGWCGS      49
Dm_AHB62682.1     -------------MRNWVLL-LLCISPFFSRTLAEQCGSQAGGALCP-------------      33
Db_QHN63861.1     -------------MRICILL-LLCVSLFLSGALAEQCGYQAGGAVCP-------------      33
Da_BAR13254.1     ------------MMKISLLL-LLFVVPLLSGTSAQQCGYQAGGALCP-------------      34
Dr_AMM76171.1     -------------MSITIML-LLCLAPLLSGTCAVQCGSEVGGALCP-------------      33
Dt_ChitI          -------------MSITIML-LLCLAPLLSGTCAVQCGSEVGGALCP-------------      33
Dc_AZZ09188.1     -------------MRITVLL-LLCVAPLLSGTYAVQCGSEVGGALCP-------------      33
Ds_00014776-RA    -------------MRITILL-LLCVAPLLSGTYAVQCGSEVGGALCP-------------      33

Cf_GAV79313.1     -----------------AQNCGCAANLCCSQYGYCGSDYQYCG-PGCQEGP---------      59
At_NP_566426.2    ---------------------------CCSEFGWCGNTEPYCKQPGCQSQCTPGGTPP--      80
Av_00023349-RA    TSAYCGGCQSQCKCGSQAGGVLCPNGLCCSRYGWCGSTSAYCG-AGCQSQCGPPPPPQTS      108
Dm_AHB62682.1     ------------------------NGLCCSQYGWCGTTSAYCG-AGCQSQCGGSS-----      63
Db_QHN63861.1     ------------------------NGLCCSQYGYCGTTSAYCG-SGCQSQCGGGSPPPSP      68
Da_BAR13254.1     ------------------------NGLCCSKYGYCGTTSAYCG-DGCQSQCGGSSPPPAP      69
Dr_AMM76171.1     ------------------------NGLCCSKYGYCGTTSAYCG-PGCQSQCGGSSPPPAP      68
Dt_ChitI          ------------------------NGLCCSKYGYCGTTSAYCG-PGCQSQCGGSSPPPAP      68
Dc_AZZ09188.1     ------------------------NGLCCSKYGYCGTTSAYCG-PGCQSQCGGSSPPPAP      68
Ds_00014776-RA    ------------------------NGLCCSKYGYCGTTSAYCG-PGCQSQCGGSSPPPAP      68

Cf_GAV79313.1     ------CDAPSSGSSVSDIVTGNFFNGII-GQASSSCEGKNFYTRGAFLTAANYYSGFAT      112
At_NP_566426.2    ----------GPTGDLSGIISSSQFDDMLKHRNDAACPARGFYTYNAFITAAKSFPGFGT      130
Av_00023349-RA    STPK--PP-PPSSGSVSSIITAALFEQFLLHRNDASCPAHGFYTYNAFIQAASFFPGFGT      165
Dm_AHB62682.1     ------PPSPSGGGDVGSIVTNEIFNQMLLHRNDNACPANGFYTYNAFIEAARSFPGFGT      117
Db_QHN63861.1     P----LPPSPSGGGDVSSIITSQIFNEMLLHRNDNACPANGFYTYDAFIQAARSFPGFGT      124
Da_BAR13254.1     P----SPPSPSGGGDVSSIITSQIFNQMLLHRNDNACPANGFYSYQAFINAARKFSGFGT      125
Dr_AMM76171.1     PSPTPSPPSPSGGGDVSSIITSQIFNQMLLHRNDNACPANGFYSYQAFLDAARKFSGFGT      128
Dt_ChitI          PSPTPSPPSPSGGGDVSSIITSQIFNQMLLHRNDNACPANGFYSYQAFLDAARKFSGFGT      128
Dc_AZZ09188.1     PSPTPSPPSPSGGGDVSSIITSQIFNQMLLHRNDNACPAHGFYSYQAFLDAARKFTGFGT      128
Ds_00014776-RA    PSPTPSPPSPSGGGDVSSIITSQIFNQMLLHRNDNACPANGFYSYQAFLDAARKFSGFGT      128
```

```
                                             I                       II           III
Cf_GAV79313.1       GGSADDSKREVAAYFAHVTHETG-------------FFCYKREID--KSTYCASSAQWPC      157
At_NP_566426.2      TGDTATRKKEVAAFFGQTSHETTGGWATAPDGPYSWGYCFKQEQ-NPASDYCEPSATWPC      189
Av_00023349-RA      TGNATTQKKEIAAFFGQTSHETTGGWPTAPDGPYAWGYCFLTEQGNPLPTYCTPSTTYPC      225
Dm_AHB62682.1       TGDVNTQRKELAAFFGQTSHETTGGWPTAPDGPYAWGYCFKQEQGNP-PSYCEPSTAYPC      176
Db_QHN63861.1       TGDINTRKKELAAFFGQTSHETTGGWPTAPDGPYAWGYCFKEEQGNP-GSYCVASSTYPC      183
Da_BAR13254.1       TGDTNTRKNELAAFFGQTSHETTGGWSTAPDGPYAWGYCFKQEQGNP-GDYCAPSSTYPC      184
Dr_AMM76171.1       TGDINTRKKELAAFFGQTSHETTGGWPTAPDGPYAWGYCFKQEQGNP-GDHCVQSSSYPC      187
Dt_ChitI            TGDINTRKKELAAFFGQTSHETTGGWPTAPDGPYAWGYCFKQEQGNP-GDYCVQSSSYPC      187
Dc_AZZ09188.1       TGDINTRKRELAAFFGQTSHETTGGWPTAPDGPYAWGYCFKQEQGNP-GDYCVQSSTYPC      187
Ds_00014776-RA      TGDINTQKKELAAFFGQTSHETTGGWPTAPDGPYAWGYCFKQEQGNP-GDYCVQSSTYPC      187

                                 IV                                        V
Cf_GAV79313.1       APGQQYYGRGPLQISWNYNYGPAGQSIGFDGLNSPDTVATDVVISFRTSLWFWMNNVH--      215
At_NP_566426.2      ASGKRYYGRGPMQLSWNYNYGLCGRAIGVDLLNNPDLVANDAVIAFKAAIWFWMTAQPPK      249
Av_00023349-RA      APGKSYYGRGPIQISHNYNYGLCGNSIGQPLLANPDLVASDAVISFKTAIWFWMTPQGNK      285
Dm_AHB62682.1       APGKSYYGRGPIQISYNYNYGQCGDSIGQPLLANPDLVADDALISFETAIWFWMTPQGDK      236
Db_QHN63861.1       APGKKYYGRGPIQISYNYNYGQCGAAINQPLLANPDLVASNADISFETAIWFWMTPQGNK      243
Da_BAR13254.1       APGQKYYGRGPIQISYNYNYGLCGAAINQPLLSNPGLVASDADISFETAIWFWMTPQGNK      244
Dr_AMM76171.1       APGKKYYGRGPIQISYNYNYGQCGAAINQPLLSNPDLVASNAGVSFETAIWFWMTPQGSK      247
Dt_ChitI            APGKKYYGRGPIQISYNYNYGQCGAAINQPLLSNPDLVASNAGVSFETAIWFWMTPQGSK      247
Dc_AZZ09188.1       APGKKYYGRGPIQISYNYNYGQCGAAINQPLLSNPDLVASNADVSFETAIWFWMTPQGSK      247
Ds_00014776-RA      APGKKYYGRGPIQISYNYNYGQCGAAINQPLLSNPGLVASNADISFETAIWFWMTPQGSK      247

                                                     VI           VII
Cf_GAV79313.1       ----GVM---------------NQGFGATIRAI-NSIECNGGNSAAVQKRVTYYTDYCNQ      255
At_NP_566426.2      PSCHAVIAGQWQPSDADRAAGRLPGYGVITNIINGGLECGRGQDGRVADRIGFYQRYCNI      309
Av_00023349-RA      PSCHDVITGNWTPSPTDLAANRLPGYGVLTNIINGGLECGIGPDARVADRIGFYNRYSQI      345
Dm_AHB62682.1       PSCHDVITGNWSPSSADQAAGRLPGYGVITNIINGGVECGKGQDASVADRIGFYTRYCNI      296
Db_QHN63861.1       PSCHAVITGQWSPSSADQAAGRVPGYGVITNIINGGIECGKGQNAEAANRIGFYERYCSL      303
Da_BAR13254.1       PSCHAVATGQWNPSSADQAAGRVPGYGVITNIINGGVECGQGEKAEVADRIGFYQRYCSI      304
Dr_AMM76171.1       PSCHAVATGQWTPSAADQAAGRVPGYGVITNIINGGVECGKGTVPQVADRIGFYQRYCSI      307
Dt_ChitI            PSCHAVATGQWTPSAADQAAGRVPGYGVITNIINGGVECGKGIVPQVADRIGFYQRYCSI      307
Dc_AZZ09188.1       PSCHAVATGQWTPSVADQAAGRVPGYGVITNIINGGVECGKGTVPQVADRIGFYQRYCSI      307
Ds_00014776-RA      PSCHAVATGQWTPSAADQAAGRVPGYGVITNIINGGVECGKGTVPQVADRIGFYQRYCSI      307

Cf_GAV79313.1       FGVSPGGNLYC---------------  266
At_NP_566426.2      FGVNPGGNLDCYNQRSFVNGLLEAAI  335
Av_00023349-RA      LGVDPGPNLDCYTQTSF---------  362
Dm_AHB62682.1       LGVNPGNNLDCYNQAPFS--------  314
Db_QHN63861.1       LGISPGNNLDCNNQRPFS--------  321
Da_BAR13254.1       FGISPGQNLDCYNQRPFS--------  322
Dr_AMM76171.1       VGISPGGNLDCYNQRPFS--------  325
Dt_ChitI            VGISPGGNLDCYNQRPFS--------  325
Dc_AZZ09188.1       MGIAPGGNLGCYNQRPFS--------  325
Ds_00014776-RA      MGISPGGNLDCYNQRPFS--------  325
```

(c)

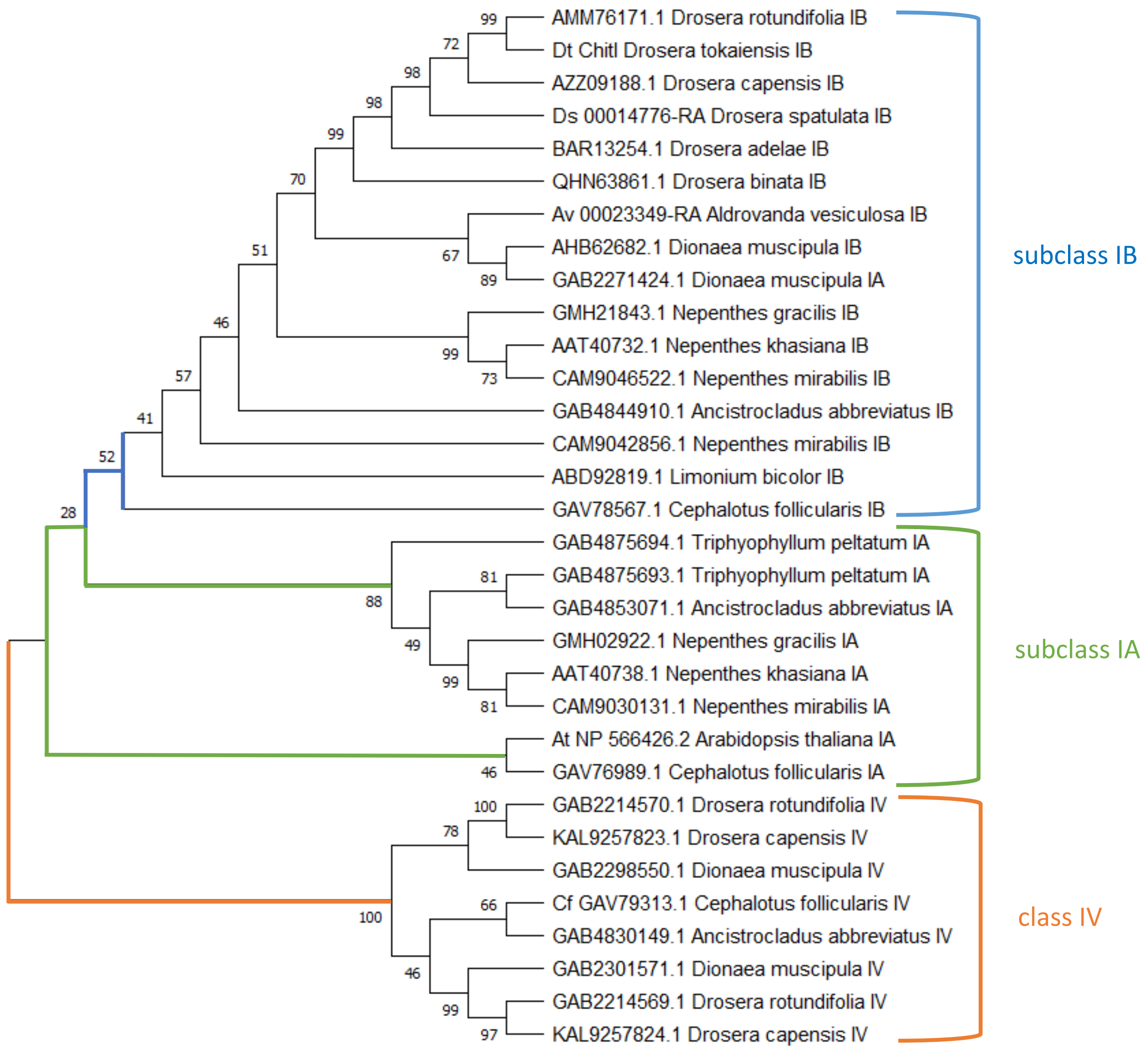

Figure S1: Multiple sequence alignment of GH19 (class I) chitinases included in the study. (**a**) Class I chitinases of the Droseraceae family in FASTA format were aligned using Clustal Omega and visualized with JalView. Color intensity represents percentage identity. Av_00023349-RA, *A. vesiculosa*; Dm_AHB62682.1, *D. muscipula*; Db_QHN63861.1, *D. binata*; Da_BAR13254.1, *D. adelae*; Dr_AMN76171.1, *D. rotundifolia*; Dt_ChitI/1-325, *D. tokaiensis*; Dc_AZZ09188.1, *D. capensis*; Ds_00014776-RA, *D. spatulata*. (**b**) Homologous chitinase amino acid sequences and their consecutive domains were aligned using Clustal Omega. Yellow highlight: Signal peptide; Green highlight: Chitin-binding cysteine-rich domain; Magenta highlight: Proline-rich hinge; Cyan highlight: Catalytic domain; Gray highlight: Active sites I−VII; Red highlight: C-terminal extension. At_NP_566426.2, *A. thaliana*; Cf_ GAV79313.1, *C. follicularis*. (**c**) Maximum-likelihood phylogeny of representative plant GH19 chitinases (including carnivorous Droseraceae, Nepenthaceae, and Cephalotaceae; the part-time carnivorous *Triphyophyllum*; the genus *Ancistrocladus* representing lost carnivory; non-carnivorous *Limonium bicolor* that secretes mucilage from stalked and sessile glands; and the non-carnivorous model plant *Arabidopsis thaliana*). The tree was inferred from 32 amino acid sequences using the WAG+G model in MEGA X. Bootstrap support values based on 1,000 replicates are shown at the nodes. Representative class IV GH19 chitinases were used as the outgroup.

>Drosera adelae LC037409.2
TCGCTCATGAATTTGCAAGGCCATGCACACGTTATGTCCTCTTGTCAAACTCGACTATATTTTGGAACTCACAACAA
CCTGCAAGACACGCACACACTTGTGTTCTCAACCCGAACGTGCTCTCAAAACTATGGATTTTGCACAAAGTTTAACC
TTTCCCTAGGTTGTTTTCTGAGAAGACAGGTTGGCCGTGCTAAGTGAAAAAAAAAAAAAAATCTGGTGAAATAGTTTT
TGCTTGGGACTAATTTGTGTTAAAACTTGTTCGAGTATACCCTTAAATGTAGGGTAGAATTAGTCAATTTGTGAAGC
TCTGGAATTCGGATATGCCGTGATATATGATGCGTATTCCTTCTTAGGTTTGGTATTATCTATACTTGCTCATAGAC
CTTGGCCTCTTAGCTATAGCATTTAGGCTTCATGGATTCTCTTAATTTTGTTGTTACAGATTTAGGATAGCTGTGGG
ATGTTTTATTTCAGAGTGTAATTGTAAAACTAATAAGACTTGTTCAATGGTACCTCTTTTCATCAATTATTTCATGC
GAATATAAAAGTTTATTTTCTTAATAAACATGTCCCACAGGCCGCAAGACAACTTGAATACATGCTAACTTATGGTG
ACGAACAGATATGCTTAATAATTTAGTTACAGTAGTAAAAATTGAGTTCTGGTTCAGATATTATTTTCGAATTGTCG
TCTTATGATCAGCATATCGAGGTTATATACATCATGAAACATAAGGGGTTATGGATTGTTATCCGAGCTGCGAGAAA
ATTTCTAAAGAGAGTTATGAAGGGATAATATTTATGACTTTTCCAGGAAGATATGTATGGTGGTTTATGATCATGAT
GGTCCAAACTGTGGCAAAATTCTTCTCTAGGTTGCTATTGGTATTTCTTGAAATGAACTTACTAAAAACTTGAAGGA
TGATTTTCCGTCTGCCTAAATCCGATCTTTCTAAAAACTTGAAGGATGATTTTCCGTCTGCTTAAATCCGATCTTTC
TCATGGTATGTATGCCTTGTTATTTATTTATTAATTTACTTCTGAAGTATTATCACAGTATTGTTGTGTTTCCTGCA
GGTATACAGTGACCATATAACAGGAAGATTATGTTTGCCTTTTTAGTTCTCATTTTTCATGGGCAATCCAATTTTAT
AACTTCTGGCTTAAGAGCAACCTCTAAATTTTTCCTTGGTCGGCTCGCTGTAGCTTCCAACGTAAAGATATAGCATG
ACAAATAGGTTGTTAATATTTCGGCCAACCTGTGTGATAAATTTCACTCATGCTTTGCCCTCCTAGTGATTTCATTG
ACCATAGGATCCTAATACAGCATAAAAAATAGTAAGCATTGTCCATGGTCCATGAAATCTATAGATTATGAAATTGT
CATTAGAAGACTAATTCAATCGAAAATCCCGTGCAGGAAGTCCCTATTTATACACCTCCTGTATCCTAACTTTCACC
AGGTCATAATAAAAAAAAAATACACGGGATTAAAAAAC

>Drosera binata MN481117.1
AAGTAAAAGGATAAATATGGACTATGGTGCTGGGATGGTGTTTACTGGTGATTAGTATGTATTCTATTTCGCCACAT
GTTGCTTATCGAAATTGTTTAGATATGAATTAATCATTCTTATCGAAGTTGTTGCCCTTAATGCAGGGTAGAAATAG
TCAATGAAGGAAGTTACTTGAATTCGGATATTCACTGGCACATGATGCTTATTCCTCCTTGGTTTGGTGCCCATCTA
TAGTGAGTCCCTTCTAGACCTTGGCCTTATAGCCTTTGGATTATCGTTTCTCATATCTTTGTTGTTTGAAAGATCTA
AACACTAAATTGTCACATTAATTTATGCTGAAATTCGTTGTGGAAATCGTAAAAACAAATAGAACTTGTTACGTGTT
ACTTCTACTTTACTATGGTTATGGGGGAATTTCCTGCCTTTTCTGATATACATGGCATTTTGACTATAAAAGATAAA
TCTCCAGTCAATGCTTATGTTTTTTATGAATAAACAAGTGCTACAATGCAACTTGGTGCAAACTTGACTATATATGA
AACATGTCGTGATGATCATTTATGGGACTGCTGTGTTCTTTGCACTCTCCCTCAGAAACCCTCTATTTGGTTAATGG
ATTGAGGATGCATTTGGTTAAAACACATTGATCATAGATTTTAGCCTATCATAGTGAGAATTTCCTTAAGTGTACTG
AAAATGTATGTTCCGTAGCATTCATTAACTAATAAAGGGTGAGGGAAAAGTGTGAAGGTTTGAGATCAGGAAAGGTT
CGAACTTCATGTTTGGAACAGTGAAACTTTGAAGTATTCTAAGAGGAACCATACATTTGCAGAAGCGTAAAGCTGGT
CCATGCTCCATATTTGATCAAATACGTATGAGTTCACCATATATTGTGGCGAAATCTCAAAAAACTCAGAAATTGTC
TCTAGTACTTCCTTGCTGGAACTTGCTAGCATTTACTATCTTAGACCTCTTCTAAGCTCAACGGATGTCCACTTGAA
ATATTCTCAACATTTCATCTAACATTTTCATTTTTCTCATGTTTCTTTTCAATTTTTTTTTTAATTTTGATAATATG
CAGCTATAATTGAGTTTTTCAGTAATGATACGATTAGATTGTGCTATAGGAGAATTCCCTCTTAAATTTTCGCTTTG
CTCACATATGTTCTGTGACTATTTCAATTTGTACCTACAACCGAGACCTGTACTTGCCTTGTTCGACGCTATGAAGT
TTCCACCTAAACATAGGGTGAGAAATGGTTAGGTAATATTTCTGCCAACTTTGCGCCACAAATTTGTACATTTGCCC
TCCTAGCGATTTCATTGACTATAGGATGCTAATTATCGAGCAATGTGCATGGTCCATGAAATCTTTTGATTATGAAA
TGGTCATTGCAGGACTTATTCAACTCAAACATCTCAGCTGGGAACCCCTATAAATACCCTTGTAAAGTATCTATGCA
AGTCACAATCACATAATATACTACAAACATCATAACC

>Drosera capensis MK093978.1 & LIEC02011376.1(scaffold 01011500.1; region
18,393–19,687 bp)
TTAGTCAATTTATGAAGCACTTGAACTCGGATATACCCTGACATCTTATGTGTATTCCTTCTTTGGTTGGTACTATA
TAGACTTCCTTATAGACCTTGGCCTCTTCCTCTTTGGTTGGTTTAGATTCTCATATCTTTGTTGTTCGAAAGACCTA
AGATCTAATTTCCACGCTGCAAATTCAGGATAGCGTTGGGGTGCTGTAGTTCATAAGGGAATTGTAAAACAGATAAG
ACTTGTTCAATGGTATGGTGCCTCTCGTCATTAAGTATTTGTTTTGAATTCTAGAATAGATGAACCTATGGATTGAA
GTTTATGTTCTAAATAAACATGTTCTGCAAGAAAAACTAAAATATGTGTGGTAACTCAAGCAAAACGAACATTTGCT

GATAACCATACATGCTTATTAGAGTAGGAAAATTGCAGTATCATTTTGAATCTGTCTATCTGATTGGCATATCTAGG
ATATACTTAGTAAAAACTATGGCGTGAGCCGCCTTTAGGGATCTTATGCAAGGATAATTTCTATAATACTCTGCACC
AGATATAAGTTGAGGGGATATCTGTGGTGGTTTATGATCATCATGGTCCAAAACTCCATCGTTTGGCACAATGAAAG
TATAAAGGCAAATTGGGCAAAAATATCTAATGGAAGTTTCATTTAGGTTTTAGTATCTGTTAGTAAATAATGACTTT
GTGGACTGTATATCATACGCATTTGCATTGTAATTTCATGCATATGAACTGTAAAAATCAAAATGAGTACTAAAATA
TAAATAAAATTTCTATCGGGAAAATTGTCCCAATTTGTCCCAAAATATATCAATTCCTTTAAGAGAGATGAGATTAT
TGCAGGAGTTGTGTTACGCTGGTCCATGCTCCATTTTAACCATACCACTATATCGTGGCAATGTTTTAATCTCTAGA
GAAGTTACTTCTCGAAAGGACCTTACTAATACTTCTTGAAAGGACCTTACGAAAGTCGAATTGGACAATTTTCTACT
GCTTAGCTCGGATTTTTCTCACAGTATGCCGTGTTATTTATTATTATCATTACTATTTTATGAAGCAACATCACAGT
ATTGTTGTGTTTCCAGGTATACATTAACTATGTCAGCCTTCTAGTTCTCATTTTTCCACGGGCAATCAATTGTATAT
CTTCTGGTTTAACCGACTTCTAATTTTACCTTAATCAGCTCGCTGTTTTTTCACTAAAAAAATAGTGTCACAAATAG
TTATTTAATATTTCAGCCAACCTATGTGATAAATTTNCNCTNANGNTCTGCCCTTCNAGTGATNTCATTGACCCTGG
ATTCGTATATATACAGCAGGAAAAATGTCTAGCATTGTGCATGGTCCATGAAATCTATAGATTATGAAATAGTCATT
AGAAGACTTGTTCAATTGAAAAATCTGAGCAGGAAATCCCTATATATAGACCCCATGTATCCTTTAAACTTCACCAA
GTCACAATCATAACATAAAGAATTAGTTATCAGAAAC

>Drosera rotundifolia KU516826.1 & BAAGIH010000008.1 (scaffold 8; region 25,566,514–25,567,513 bp)
ATAGACCTTGGTCTCTTAACCTTTAGGGTTTAGATTCTCATATCTTTGTTGTTCGAAAAACCTAAGATCTAATTTCA
ACGCTGCAAATTCAGGATAGCGTTGGGGTGCTGTAGTTCATAGGGGAATTGTAAAACAAATAAAACTTGTTCAATGG
CACGGTACCTCTCGGCATTAATTTTTTGTTTTGAATGTAGTTTTGAATTCTAGAATAGATGAATCTATGGATTGAAG
TTTATGTTCTAAATAAACATGTTCTGCAAGATAACCTGAATACGTGTGCTAACTCAAGCAAAAGGAACATTTGCTCA
TAAACATACATGCTTAATAACTTAATTAGAGTAGGAAAATTGCAGTACCATTTTGAATCTGTCTATCTGATTGGCGT
ATCTAGGATCTATTTAATGAAAACTAGGGTGTGACCCGCCTTTAGGGATCATCATCGGAGCTGTGAGAAAATTGCTA
AAAGAGTTATGCAAGGATAATTTCTATGACACTCTGCACCAGATATAAGTTGAGGGGATATGTATGGTGGTTTATGA
TCATCATGGTCCAAAACTCCATCGTTTGGCACAATGAAAATATAAAGCCAAATGGGGCAAAAATACCTATTGGAAGC
TTTATTTACGTTTTATTATTCGTTAGCATTCGTGAATTATAAACAATGACTGTGAACTATATATCATACGTTTTTGT
ACTGTAATTTCATTCATATGAATGGTAAAAATCAAAATGAGTACTAAAATGTAAGTAAAACTTCTATCAGGTACATT
TGTCCAATCGTCCCAAAATATGACAATTCCTTTAAGAGAGACTAGAATTACAGGAGTTGTGTTACGCTGGTCCATGC
TCCATTTTAACCAACCACACATTAATAAGCCCTATATTGTGGCGAAGTTCTAATCTCGAGAGAAGTTGTTACTAATA
CTTCTTGAAAGGACCTTACTAAATACTCAATTCATCTTGCGTCCGTTATGAAAGTCGAATTGAACGATTTTCTACTG
CTTAGCTCGGATCTTTCTCATGGTATGCCGTGTTATTTATTATTATCATTACTATTTTATCAAGGATTATCATAGTA
TTGTTGTGTTTCCAGGTATACATTAACTGTGTCAGCCCTTCTAGTTCTCATTTTTCCACGGGCAATCCAATTGTATA
TCTTCTGGTTTAACCGACCTCTAATTTTACCTTAGTCAGCTCGCTGTTGTTTCGCTGAAAAAATAGTGTCACAAATA
GTAATTTAACATTTCAGCAAACCTATGTGATAAATTTTCACTCATGCTCTGCCCTTCTAGTGATTTCATTGACCATA
GGATCCTATACATACAGCAGGAAAAATATCTAGCATTGCGCATGGTCCATGAAATCTGTAGATTATGAAATACTCAT
TAGAAGACTTGTTCAATTTAAAAATCTGAGCAGGAAATCCGTATATATAGACCCCATGTATCCTTCAAACTTCACCA
AGTTACATCACAACGTAAACAATCAGTTATCAGAAAC

>Drosera spatulata Ds_00014776-RA (https://www.biozentrum.uni-wuerzburg.de/carnivorom/resources/)
GGGCAAAAATACCTAATGGAAGCTTCATTTACGTTTTAGTATTCGTTAGTATCTGTGAACTGTACACAATGACTTGG
TGAACTGTATATATATCATACGTTTGTGCACCACAATTTCATGCATATAAACGGTAAAAATCGAAATGAGTACTAAA
ATATAAGTAAAACTTCTATTGGGTACATTTGTTCAATCGTCCCAAAATATAACAATTCCTTTAAGAGAGAATTGTAA
CGCCCCGGCCAAATTCTTGTTATCGGGTCGTCATAAAAAACCGCTCTCCTGCCTGACAACTGATCCCACAGACCAAC
ACTAATCATTCCATTGTGTTTTGGCCTCACTCGCGCACGTCCTAAGAAACTTCCCAAGATGTCACCTATCCCAGCAT
TACTGCACGTTAAGCACGCTTAACTGTGGAGTCGACTTCAAGCACCACACACAGCCCCACAACCTTCCCCCCGCTAT
GCGGCTGACCTGTACCTCTTGTCCATAAGTGCAGCACGCGGTCGCAGGGTTGCTCTAATATCATAATGTAACGCCCC
GGCCAAATCCTTGCTACCGGGTCGTCACCAAGAACCGCTCTTCTGGATAAGGAATGGCCACACATACCAACAAGAGT
CATTACAGTGCGTTTTTACCTCACTCGCGCACGTCCTGAGAAACTTCCCAGGATGTCACCCATTCCAGCATTACTCC
ACGTTAAGCACGATTAACTGCGGAGTTCTAAAGTAATGGGATACCGAAAAGGAAGGTGCACCTTGTTGATATAGATA

GTATTATTAAGCCATTTAAGTATTTTCTTGGGGTATTACAAGAATAGATAATACAGGAGTTGTGTTACGCTGGTCCA
TGCTCCATTTTAACCAACCACACATTAATTCGCCTTATATTGTGGCGAAGTTTTAATCTCCAGAGAAGTTGTTACTA
ATACTTCTTGAAAGGACCTTACTAAATACTCAATTCAGCTTGCGTCCTTTATGAAAGTCGAATTGGACGATTTTCTA
CTGCTTAGCTCGGATCTTTCTCATAGTATGCCGTGTTATTTATTATTATCATTACTATTTTATGAAGCATTATCACA
GTATTGTTGTGTTTCCAGGTATACATTAACTGTGTCAGCCCTTCTAGTTCTCATTTTTCCACGGGCAATCCAATTGT
ATATCTTCTGGTTTAACCGACCTCTAATTTTACCTTAGCTCGCTGTCGTTTCACTAAAAAATTAGTGTCACAAATAG
TTATTTAACATTTCAGCCAACCTATGTGATGAATTTTCACTCATGCTCTGCCCTTCTAGTGATTTCATTGACCATAG
GATCCTATATATACAGCAGGAAAAATATCTAGCATTGTGCATGGTCCGTGAAATCTATTGATTATGAAATACTCATT
AGAAGACTTGTTCATTTGAAAAATCTGAGCAGGAAATCCCTATATATAGACCCCATGTATCCTTCAAACTTCACCAA
GTCACAATCACAACATAAACAATTCGTTATCAGAAAC

>Drosera tokaiensis JAOYUI010483990.1 (region 592–2,091 bp)
ATAGACCTTGGTCTCTTAACCTTTAGGGTTTAGATTCTCATATCTTTGTTGTTCGAAAAACCTAAGATCTAATTTCA
ACGCTGCAAATTCAGGATAGCGTTGGGGTGCTGTAGTTCATAGGGGAATTGTAAAACAAATAAAACTTGTTCAATGG
CACGGTACCTCTCGGCATTAATTTTTTGTTTTGAATGTAGTTTTGAATTCTAGAATAGATGAATCTATGGATTGAAG
TTTATGTTCTAAATAAACATGTTCTGCAAGATAACCTGAATACGTGTGCTAACTCAAGCAAAAGGAACATTTGCTCA
TAAACATACATGCTTAATAACTTAATTAGAGTAGGAAAATTGCAGTACCATTTTGAATCTGTCTATCTGATTGGCGT
ATCTAGGATCTATTTAATGAAAACTAGGGTGTGACCCGCCTTTAGGGATCATCATCGGAGCTGTGAGAAAATTGCTA
AAAGAGTTATGCAAGGATAATTTCTATGACACTCTGCACCAGATATAAGTTGAGGGGATATGTATGGTGGTTTATGA
TCATCATGGTCCAAAACTCCATCGTTTGGCACAATGAAAATATAAAGCCAAATGGGGCAAAAATACCTATTGGAAGC
TTTATTTACGTTTTATTATTCGTTAGCATTCGTGAATTATAAACAATGACTGTGAACTATATATCATACGTTTTTGT
ACTGTAATTTCATTCATATGAATGGTAAAAATCAAAATGAGTACTAAAATGTAAGTAAAACTTCTATCAGATACATT
TGTCCAATCGTCCCAAAATATGACAATTCCTTTAAGAGAGACTAGAATTACAGGAGTTGTGTTACGCTGGTCCATGC
TCCATTTTAACCAACCACACATTAATAAGCCCTATATTGTGGCGAAGTTCTAATCTCGAGAGAAGTTGTTACTAATA
CTTCTTGAAAGGACCTTACTAAATACTCAATTCATCTTGCGTCCGTTATGAAAGTCGAATTGAACGATTTTCTACTG
CTTAGCTCGGATCTTTCTCATGGTATGTCGTGTTATTTATTATTATCATTACTATTTTATCAAGGATTATCACAGTA
TTGTTGTGTTTCCAGGTATACATTAACTGTGTCAGCCCTTCTAGTTCTCATTTTTCCACGGGCAATCCAATTGTATA
TCTTCTGATTTAACCGACCTCTAATTTTACCTTAGTCAGCTCGCTGTTGTTTCGCTGAAAAAATAGTGTCACAAATA
GTAATTTAACATTTCAGCAAACCTATGTGATAAATTTTCACTCATGCTCTGCCCTTCTAGTGATTTCATTGACCATA
GGATCCTATACATACAGCAGGAAAAATATCTAGCATTGCGCATGGTCCATGAAATCTGTAGATTATGAAATACTCAT
TAGAAGACTTGTTCAATTTAAAAATCTGAGCAGGAAATCCGTATATATAGACCCCATGTATCCTTCAAACTTCACCA
AGTTACATCACAACGTAAACAATCAGTTATCAGAAAC

>Dionaea muscipula KF597524.1 & JAOWBR010230478.1 (region 5,222-6,239 bp)
CTAATTAATCAGCTCCTCATGCATGGATCCGATCCAAGTGAGTCCCCTCTCATCTCTGCTCCCCCATCTTCGTTTTC
TACCCCACTCAATTTCTCTATCGTCATGATCTCGGATCCCCTAGTAAATTAGGGTCCCGGCATTGTGAATCAATCAG
TCTTGCAATGTCCGTGATCGGTTTTCAGATTAAAGATCTCGTTCCCGTTTAGATTTGGAACTTCTATCGTTTCGACT
TGATCTGATGCGTTTTCGTTTTGGTTGCTGGAATTTGTCCACCATTGGCGTGTGTCTTCTGAATCTTGATGTGTAAT
TGATTTGGGATTAATTTTAGTCTGTGTCATATTCGTTGGATATAAGGTGATGTGGTGGAAAAATTATTCATGGAGTG
CGAATTTGTTCTAGTGAATAGTGATTACTTGGGGAAGTAATTACAGTGGTGGTAATCAGAAGCCCAAAACATTCTAG
TAAATGAGTCGGTAATGAAATTGGTTTCCGAATTGAAACTGAGTTGAAATTTTGAAAATGTGTTTGGAATTGGAAAA
CAACGATTGTTTGGGTAGGATTAGATTTTTAGGGTTGAAATATTTTTCCTCTGCTTCATGCAAGTAATCAATTTGAA
GACTTCCACACAGCGTTATTAAGGAACATGTGCAAAATGAACATGTATATTCGAGAGCCTATTTAAAGGAGAGGAAG
CTATATCTGGTGATGGTCCAGGCAACATACATACTAGGAAAATTCAAGAAGTCTCTCCAGAGGTATCAGACATCTTG
AAGAGTGGCCTTGAATTGTTACATAATTGATAACACGATTCATCGCGTAATAAATGTTGACTATGCCTCCTCAAAAA
GCTGAATTTCGTAAACCGAGAGTTCGAGTATAGAAATTTAATATACCTTTGTAGCTTGACCTGGATGCATAACCCAC
GAGGGTTATCTTATACCTCTTTTAGGAATTCGAAGTGATATAGCATGATAGCAGCGAAGTGATATTGGTAAATTATA
GTAAGTTGTTACCGTTTATAGCCAAAAAATAACTTTGGATTGACAAATTTGTCTGATTATATTGGAGTTGCTCTTTA
TGTAATGGAACATTGTCTTCCTTTAAAATTCACCCCCTTTTATGGACAATTTGAATGCATTTGTTTTGAGATTACGT
CACGCTATCGAGGTCGATAGGTATATTTTGTTAGCACCTAGGGAACCTGACGGGGGCTAGTAGCCAAACAAGGTGGA
CTATATATAGGAACAGCTACGATTGATTAGTATTTTAGCCCACTCTATGCAATCAATAAATAGATGTAATCTTATAT

GTGAAGCAATGTACATGGGCCATGAAATTCATCGCTGTACTAAATGGTCAACAAAGACTTGTTCAATCACTTACCGA
AAAAAAGACTTGCTCAACTGAAAAACTCAGCAGGAAACCCTATATATACCCCTGTATCCATGAATTGGTTTTGCAAA
GTCACAATTAGAAAATACACAACAAAAGGGAACAAGC

>Aldrovanda vesiculosa Av_00023349-RA (https://www.biozentrum.uni-wuerzburg.de/carnivorom/resources/)
AGACGACACGGCACATGAATAAACGGCACAAACACATTAATGGTTTAATGGATTTTTTAAAAACAGTGTCATGCCTT
ATTAGAACCGTATCGTGTCGTGTTATGCCCGACTAGAACCGTTCCGTGTCCAACTATAACTGTGTCGTGTTGTGTCT
ATCACATCCGTGTTGTGCCATGCCAGATTCAAATTGTGTCGTGCCATGCTCATACCAACCCCTATTCGTGTTGTGCT
CTACTGCGCTCATGTCGCATACTTGACTTGTTCGGCTCGATTATACTTGTGTTTTACCCTTATCATATCAACTTTTG
TGTCGTGTCATGCCCGTTTATTATCGTCACTCGTGTCGTGTTATGTGTCAGTTAAAGTTGTGTCGTGCTCGTGTTAG
ATGACATAAACACAAACGTGTCGTGTCATGTCATGCTCATACCAGTGCGGCCCACTTTATACACCTACAAGCAATCA
TAAGTGTGACTTAAAAGAACTAACAATCTCTAGAGATGACTTGTGGAATACAAGATAATCCCCTTACATTCCCATAC
GATGATAGTAGTGACGATCACGACAACTAATCAATTCACAAATCAAAGCAGCTTATCAATTAGCTTAATTTTTATAA
ATGATTTGTGCTTTTAGTCTTTTACACACAAATCAAAGGCAGCTTATCAATTAACGTAATCGTTGTAAATGATTTGT
GGTTTTAGTAATTTACGCACGTACTTATATATTTTCGGTATTGTTTGGTAACTACATGAATATTTTATAATTCTACA
AGTTTTTTCTTTTTCTTTTAAGCTTTCCATTTGTCCATTGGAAAATTTGTAACTCATACAACTATATCTACCCAACC
CATAATGATTATGTCATTTGGTTTTCCAATCATGTTAATGTGTGTTTTTGACATTAGTCGATTTATACTTGTTGGTC
ATTGGTTTAAACGAGATGGATGACTTCGAGTTTCAAATTAAAAAAATCTACTTAATAGATATACAAATAAAATATTC
ATTTGTTTTTTGTTTTAGGTCTCATAGTTACGACACAATTAAAAATATTCATTATGTTAATATATATATATATATAT
GTATTAGAATTGGTTGGTGGGGAAGTATTTATTCTAAGTTAAATGTATTATGCTATGATCCTCCAGTGCCTACATAA
ATTGTGAGCAAAACTTTTGGCCACAAAGAAAGTCCTCTTTAAAAAAAAAACAAACTGGAAGGACTTTTGATCAGCCA
CGAGGAGAGTCTTTTTTTATGAAAAAAAAACAAATTGGAAGGTCTGATTATATATATATATATATATATATATATTA
AAAAAAACAAACTGGGAGGACTTTTGGTCAGCCACAAGGAAAGTCTTTTTCTAAGAAAAAAACAAACTGGAAGGACT
TCTGATCATCCACGAATTGGAAGGACTTCTGATTAGCCACATGCTATATATACCATCCCGAAGTCACGATCAGAAGA
CACACGTACAACAAATCACTAGAAAAAGTGTAGAATC

>Cephalotus follicularis Cfol_v3_22778 & BDDD01001956.1 (region 179,247-180,746 bp)
AAAGCTATGGCGGCTTCGTTTTTTAATCCCAAGTTTACCCTAAAATTCACAAGTTTCTTTTGATGTTATCACTGATC
CATCGTATATTTGCACTTTCTTTGAGCATATTCTGCTTTGAATTAGACATCAATCATTAGCAATGAGAGAACCCAAA
AAAATTGTAGTATTGAAGCCTAAAATCAACTATTCATAAAGTGAGATACTCTATACTTTATCTATCAAACTTGATTT
CCTGCTCTTTCTTGAGTTGTATATAAATGATTCTCTTTCGGTTAAAACATTAATATACATGTTTAAGAATGTTTCTT
TTCTTGGAATGGGACGATATGGGATGCCATACACGTCAAATTAAGTTGAAGATGAACTCAATTTCAATTTTAGAATG
CAACTGCTTATTAGCTAATTCTATGAAAATTTTACAGATACCATCAACTATATATATATATATATATATATATCAAAGA
GATCATCCCCATTAATTTACAAAATTCTACCAACACATTCCTAAACACCGTACATACACAATTTAGATGTTGTAAGG
ATCCTTCTTTTCCCATTGAATGCAAACTCTAAACTCTTTTCTTTATAGGAAAATTTGAACCCCTTCTTGCTGTAGAG
AAGCTCTATGAGTTTTAACATTCGACCAAACCTAAAAAGAATTTTGTATAACTACTTCTCAAAGTAGTAGTAAATGT
TGCTTTTACTATACATGCACCATAAAAAATAAGAGGATTATAAACAGTTAATTTTGTTGTTTGTTACTGAAAAATTT
TGCAATAAAAACTGTTACTAATGCTTGTCACGAATAATTAAATTTCTTACAATAACGGGAGTTACCTCTTGAACTCA
AAGCAGACAATATCTCACAGGATTTAGGTATGATCGTTACAAATGGCATCAGAGATGACTCAATACAACTTTTATGT
AAGATACGTAGTCATGCTGGGCTGGTCACGAGCCTGTCGTGGTATTTGGATATGTTTGGTTTGTCTCCTGACGAGGA
TGTCAGGGATTAAACGAGAGAGTTTGTCATGACCCGCATCCTATATCGGAAATATGTTGAGAAGATCTTTAATTATA
TGTGAAAACCCTTTTTAAACTTATGAGACGCTTTTTGAGGACAAAATCGTGTGGGACAAAGTGTCACGTATGAGATA
TGGAACCACCTCTTGAACTCAAAACGAACAATACCTCACCAGATTTAGTCGGAGTTGTTACATATATTCCCCTATAA
TAGCATTCATGTACATAAAGTTAAAAGATTTAATAGCCGCCTACATGTTCTTCTTTTCAAACTTATTGATAATATAA
CACTATAAGATAAGATGGGAAGCTTAAATTAGACTACGCAATTGAATAAGTGAAAAAGCATTGACCAAGACAAATTA
CAATAACAAAACAAATTGTGTTTGTCACCATCAACTCGTTGGCTATATATACCCACCCTTCCAAATTGCTATAGCAC
ATCAACAAAATTAATTACAAACACAAACAAAAAAAAA

>Arabidopsis thaliana HCHIB or At3g12500 & NC_003074.8 (region 3,963,985–3,965,484 bp)
TATAATCTAAACATTTTAGCTAATAACATAGGAGTGCACCATATTTATTTAATAGGAGAATGTACTATATGACCTGA
ATGCACTTTTACTCAAATCCACCAAAAAAGTCTTGCAAAAATACTATAGACCCGAATGTACCATATTTTTTTATGCT
AATTTTTCTGTAATTAAGATTTTAATTGATTTCGTATATAAAATATTAAATTGTTTTACGAACTTATTTGTTTATAA
TTTGGTAACAGATAGATATTTGAATATTCGGTATATTTTGCTTCAGTTTAGGAAATCTTTGATGACCCGTCTTTAAA
TCCAATAAGCCCTATAATTAAGAGATGAATATTGTCGTTAAATGATTTTGGTAATCTTAATGATGTCATTAAAAGAT
TCTATTTTTGATTAAAATTTTCTAATGGTATTGTCATGTAATTAATTACAAAAATTAAGATTACATTTAAAATGTAT
TTTGTAGGATTTGCCAATATATTCACATTACAATATTATTTTTTTCCATGGTCGATTATTTCTACCCGAAAGAGACG
GCAACGCAGCCCACAGTGAGATTGGAGATTAGAGATTCAGTCAACGATGATTAACGTTACTTTTCATAGTTAGTACA
TAAGAATAGCACGCAGGTTTCGTCGAGGTTTTGGATTTTATATAGTTTGTAAACATGACACGTACTTTGGATAAAAC
ATTATGAAATAGGGAAAGTCATCATGAGTAGTTGGAATAATGAATATATTAAATATTGGTATGATTTGGTCTTGAGT
CATTATATCATTACCTGGCTAATGCTATACGTTACCTTATAATGAATTCTAAATGTCTCAATTGCTTTTTGAAAAAG
TTGATCACGAACCCGCCGCCTCATATTCATAATTAAAGTTAAATTTTGTTTTTGTCAACATTGATCATAGTTAAATA
TTTAGACACGTCTATTTTTATTTTCTCCACGTTATACATTTTCTTGACTATTCATGCAACTCTTGTGATCTTGTCTT
AGATATAGTCAAGTATTGTGTATACATCATAGGGAATATCCGAATATGTGTGTGTTTGATTAATTAGTGCACACTAG
CTCAGATCATCGATTAGTCCGCAACTCGAGTAAACGTGTTTTCAGACCACCCAAAATTTTACGACCTTTGTGTCCCA
CTATCTACGTCGATAGTAACTTTAGAACAAAAAACTCAAAAAGAGTGTAATTTGTTATGCTTTTGGTCTTCTAAAAT
TTGTATTCTTCTACCAACATTTAGCACAGTTTCATACATAGAAAGTTCTCATTACTTGGAATTAACATGATAATCCC
CCAAAAAAACAAATCAATATCTAACCTAGCAACAATGACGGAAGGAAAATAAATATAATCAAAAACAAACCTACCAA
ATACTGTTTGTTCCCCATAGCCACACTCCATAGACATAACAAACAATAAACTATGGAAAAAGATAGAATTTGATGTG
ATAAAACATTCATTAATTGGATCTATATATATCTTTC

Figure S2. Nucleotide sequences and their accession numbers of the analyzed GH19 chitinase gene promoters. All available genomic sequences of the Droseraceae family were included in the study: *Drosera adelae*, *Drosera binata*, *Drosera capensis*, *Drosera rotundifolia*, *Drosera spatulata*, *Drosera tokaiensis*, *Dionaea muscipula*, and *Aldrovanda vesiculosa*. Additional reference sequences include those of the carnivorous plant *Cephalotus follicularis* and the model plant species *Arabidopsis thaliana*.

Table S1. Available expression and functional evidence for the analyzed Droseraceae GH19 class I chitinases.

Species	Gene/protein	Evidence of transcription/expression in carnivorous tissue	Experimental association with digestion	Reference
Drosera adelae	BAR13254.1 / Chi1	Chi1 expression was detected almost exclusively in glandular tentacles, with most transcripts originating from tentacle heads.	Class I chitinase was identified among the major proteins of the digestive fluid.	Arai et al. 2021 [2]
Drosera binata	QHN63861.1 / DbChitI-3	Highest basal expression occurred in leaf blades bearing digestive tentacles; expression was much lower in petioles and nearly undetectable in stems, roots, and flowers.	Transcript abundance increased after treatment with gelatin, chitin, and pachyman, supporting a role in digestive responses.	Mikitova et al. 2025 [15]
Drosera capensis	AZZ09188.1 / Chit19	The coding sequence was recovered from cDNA generated from RNA isolated from leaves. Quantitative tissue-specific expression was not evaluated in the cited study.	Recombinant Chit19 showed chitinolytic activity; direct in planta digestive induction of this specific gene was not tested.	Sinelnikov et al. 2020 [24]
Drosera rotundifolia	AMM76171.1 / DrChit	DrChit was expressed predominantly in tentacles.	Expression increased following treatments mimicking prey presence, including chitin; recombinant DrChit exhibited chitinase activity.	Jopčík et al. 2017 [16]
Drosera spatulata	Ds_00014776-RA	The transcript was detected in mature leaves of non-induced plants, indicating basal expression in carnivorous leaf tissue. Prey-induced upregulation of this specific gene has not been tested.	No direct experimental evidence for prey-induced expression or digestive function of this specific chitinase is currently available.	Pálfalvi et al. 2020 [22]
Drosera tokaiensis	Dt_ChitI	The chitinase transcript was identified in the available leaf transcriptome.	No quantitative tissue-specific expression or direct functional evidence for prey digestion is currently available.	Transcriptome accession GKWH00000000.1
Dionaea muscipula	AHB62682.1 / VF chitinase-I	Transcription of VF chitinase-I was reported to be restricted to digestive glands and enhanced by secretion-inducing stimuli.	The protein was identified in secreted digestive fluid and shown to hydrolyze chitin.	Paszota et al. 2014 [17]
Aldrovanda vesiculosa	Av_00023349-RA	The chitinase transcript was detected in transcriptomic data from plants cultivated under conditions with zooplankton available as prey. No prey-induced expression of this specific gene was demonstrated.	Direct functional evidence linking this specific chitinase to prey digestion is currently unavailable.	Pálfalvi et al. 2020 [22]

* Basal expression in carnivorous leaves has been reported for several *Drosera* class I chitinases, with increased expression after prey- or elicitor-related stimulation in experimentally characterized species. However, prey inducibility has not been tested for all genes included in this study.

Table S2. Pairwise uncorrected p-distances among the analyzed 1,500-bp chitinase promoter sequences.

	**P-DaChit**	**P-DbChit**	**P-DcChit**	**P-DrChit**	**P-DsChit**	**P-DtChit**	**P-DmChit**	**P-AvChit**	**P-CfChit**	**P-AtChit**
**P-DaChit**										
**P-DbChit**	0.46032									
**P-DcChit**	0.35276	0.37789								
**P-DrChit**	0.34242	0.38047	0.08758							
**P-DsChit**	0.38969	0.45336	0.27273	0.26885						
**P-DtChit**	0.34394	0.37879	0.08902	0.00267	0.26885					
**P-DmChit**	0.53058	0.51839	0.50368	0.51375	0.54386	0.51218				
**P-AvChit**	0.57591	0.57559	0.52766	0.52510	0.56224	0.52346	0.57415			
**P-CfChit**	0.53807	0.52680	0.51304	0.49711	0.54310	0.49711	0.58030	0.58758		
**P-AtChit**	0.50526	0.53119	0.48510	0.49094	0.54011	0.48936	0.59157	0.58733	0.58499	

* Values are uncorrected p-distances (proportion of differing nucleotide sites); gaps, missing data, and ambiguous sites were excluded by pairwise deletion.
